# Logical-continuous modelling of post-translationally regulated bistability of curli fiber expression in *Escherichia coli*

**DOI:** 10.1186/s12918-015-0183-x

**Published:** 2015-07-23

**Authors:** Kaveh Pouran Yousef, Adam Streck, Christof Schütte, Heike Siebert, Regine Hengge, Max von Kleist

**Affiliations:** Department of Mathematics and Computer Science, Freie Universität Berlin, Arnimallee 6, Berlin, 14195 Germany; Faculty of Biology/Microbiology, Humboldt Universität zu Berlin, Chausseestraße 117, Berlin, 10115 Germany

**Keywords:** Logical modelling, Stochastic modelling, Bistability, Phenotypic heterogeneity, Biofilm, C-di-GMP, *Escherichia coli*

## Abstract

**Background:**

Bacteria have developed a repertoire of signalling mechanisms that enable adaptive responses to fluctuating environmental conditions. The formation of biofilm, for example, allows persisting in times of external stresses, e.g. induced by antibiotics or a lack of nutrients. Adhesive curli fibers, the major extracellular matrix components in *Escherichia coli* biofilms, exhibit heterogeneous expression in isogenic cells exposed to identical external conditions. The dynamical mechanisms underlying this heterogeneity remain poorly understood. In this work, we elucidate the potential role of post-translational bistability as a source for this heterogeneity.

**Results:**

We introduce a structured modelling workflow combining logical network topology analysis with time-continuous deterministic and stochastic modelling. The aim is to evaluate the topological structure of the underlying signalling network and to identify and analyse model parameterisations that satisfy observations from a set of genetic knockout experiments. Our work supports the hypothesis that the phenotypic heterogeneity of curli expression in biofilm cells is induced by bistable regulation at the post-translational level. Stochastic modelling suggests diverse noise-induced switching behaviours between the stable states, depending on the expression levels of the c-di-GMP-producing (diguanylate cyclases, DGCs) and -degrading (phosphodiesterases, PDEs) enzymes and reveals the quantitative difference in stable c-di-GMP levels between distinct phenotypes. The most dominant type of behaviour is characterised by a fast switching from curli-off to curli-on with a slow switching in the reverse direction and the second most dominant type is a long-term differentiation into curli-on or curli-off cells. This behaviour may implicate an intrinsic feature of the system allowing for a fast adaptive response (curli-on) versus a slow transition to the curli-off state, in line with experimental observations.

**Conclusion:**

The combination of logical and continuous modelling enables a thorough analysis of different determinants of bistable regulation, i.e. network topology and biochemical kinetics, and allows for an incorporation of experimental data from heterogeneous sources. Our approach yields a mechanistic explanation for the phenotypic heterogeneity of curli fiber expression. Furthermore, the presented work provides a detailed insight into the interactions between the multiple DGC- and PDE-type enzymes and the role of c-di-GMP in dynamical regulation of cellular decisions.

**Electronic supplementary material:**

The online version of this article (doi:10.1186/s12918-015-0183-x) contains supplementary material, which is available to authorized users.

## Background

The ability to adapt to external inputs is a crucial property of cellular systems [1]. Bacteria, for example, can outlast antibiotic stress by forming biofilm colonies [2], or they may produce bacteriocins when sensing nutrient competition [3]. This form of acute adaptation is often encoded in the bacterial signal transduction network, which enables multiple stable states and may thus give rise to phenotypic heterogeneity [4–6].

In *Escherichia coli*, the regulatory system of amyloid curli fibers is a central component of the stress-induced biofilm formation under the control of the master regulator *σ*^s^ [7–9]. While heterogeneous all-or-nothing expression of the curli protein CsgB in isogenic wild type cells under identical environmental conditions was shown in [10, 11], subsequent work [7] allowed to infer interactions of the molecular components of the underlying signalling network. However, the ability of the suggested network to produce bistable behaviour (curli on/off) and the influence of individual molecular components on single-cell dynamics still remains to be elucidated.

Rigorous mathematical modelling may further our mechanistic understanding of this system. Two different methodological views for analysing bistability in signalling networks have been established in previous studies. Logical modelling approaches assign discrete states to different activity levels of network components and represent reactions by logical functions. The advantage of this methodology is that it allows to test a large amount of alternative model topologies using a limited amount of data, or semi-quantitative data such as promoter activities in genetic knockout strains or immunoblotting data [12, 13].

Particularly regarding asymptotic behaviour, logical analysis has been shown to be a useful tool for uncovering fundamental characteristics and functionalities of biological systems, (see e.g. [14–16]). Consequently, it is a well-suited approach for investigating bistable systems, where there is evidence that qualitative properties, in particular the network topology, are crucial determinants [17, 18]. However, the low detail resolution of such models only allows for a preliminary understanding, and the biological interpretation of the results is not always straightforward.

Time-continuous models (ODE-based or stochastic) entail more biological detail and model parameters are readily interpretable. In this approach, besides topology, the signalling network is defined by well-established reaction rate kinetics. Among others, the approaches for analysing bistability in continuous models are based on a feasibility analysis of unstable states [19] and the chemical reaction network theory [20], as e.g. applied to study bistable regulation of split histidine kinases in two-component signalling networks [21]. In analytically less tractable models, heuristic parameter search may be used, such as Monte Carlo [22] or the genetic algorithm [23]. However, an exhaustive analysis of a large space of alternative model topologies and parameterisations is often infeasible, requiring a confinement to certain regions of the model and parameter space. Thus, a drawback of the continuous modelling approach lies in the containment to a network topology and the requirement of sufficient information about the underlying biochemical reactions.

In addition to the deterministic modelling approach, time-continuous stochastic modelling [24–26] may deliver further insights into the system behaviour such as the probability of the cells to be in either of the two stable states and the noise-induced switching dynamics between the stable states [27, 28]. The high computational effort for analysing stochastic models precludes, with only few exceptions, e.g. [29], from addressing inverse problems (model and parameter estimation) at all.

**Fig. 1:**
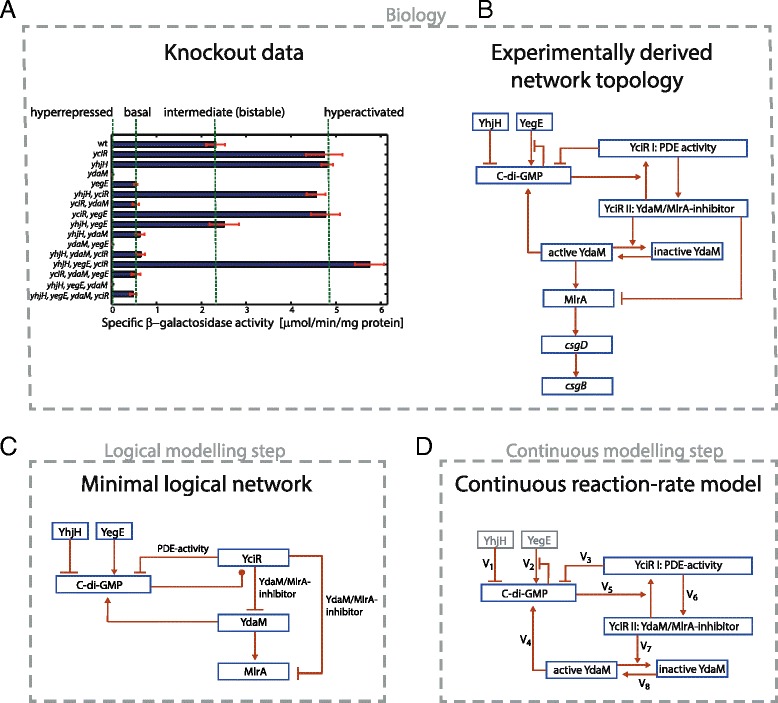
Logical-continuous modelling pipeline for bistability analysis of curli regulation in *E. coli*. **a** Expression of the curli gene *csgB* in mutants with single or multiple knockout mutations in YegE/YhjH and YdaM/YciR c-di-GMP control modules (as indicated). Derivatives of *E. coli* K-12 W3110 carrying a single copy *csgB*::*lacZ* reporter fusion were grown in LB at 28 °C for 24 hours and *β*-galactosidase activities were determined. Figure reproduced from [7] with permission. **b** Signalling network regulating the expression of curli fibers, as suggested in [7]. **c** Logical formulation of the network topology. The two different functional states of YciR are modelled by two types of interactions. Only the inhibitory effects exerted by YciR are represented by edges in the figure. Full details on the regulatory activity of YciR and the dependence of the two functional states on the activity of c-di-GMP is formalised in the Additional file 1: Figure S1c. **d** Continuous model combining kinetic reaction rates with the validated network topology

Combining the advantages of the logical and continuous modelling approaches may allow to overcome their individual limitations and thus significantly increase the computational feasibility of the analysis, while keeping a high level of detail. The different views may generate complementary insights, contributing to a more holistic understanding of the topology and the kinetics of signalling networks [30, 31]. To exploit these aspects, in this paper we introduce a hybrid modelling pipeline combining available genetic knockout data with logical, ODE-based and stochastic modelling approaches as outlined in Fig. 1a-d. Starting with a logical, constraint-based description of the available data, we generate and analyse a pool of feasible logical models. This first step yields, on the one hand, results on essential system properties on its own. On the other hand, it is used to extract a well-supported network topology for the regulatory system of amyloid curli fibers in *E. coli* (Fig. 1c), as well as parameter constraints for building a more detail-resolving ODE model. It has been shown in the context of biological modelling that properties related to asymptotic decision processes are rather robust concerning parameter perturbations up to being sustained between continuous and logical models (see e.g. [32]). Notably, steady states are rather well preserved between models as demonstrated in a number of studies [33, 34]. Based on the network topology validated in the logical modelling step, we incorporate additional kinetic information of involved reactions in order to set up a continuous reaction-rate model of the signalling system (Fig. 1d). Deriving an ODE-based model from the reaction rates enables us to estimate parameters that induce bistability compliant with experimental constraints. Finally, we generate sample trajectories from the corresponding Chemical Master Equation in order to analyse the dynamics of stochastic switching between the stable states of the system. The latter approach allows to identify alternative scenarios of the cell population splitting into curli-on (biofilm) and curli-off states, while allowing stochastic back- and forth switching or leading to ultimate differentiation, in line with biological observations [11].

## Methods

### Regulatory network of curli expression

The second messenger molecule bis-(3’-5’)-cyclic dimeric guanosine monophosphate (c-di-GMP) is the central component within the regulatory signalling network of curli fimbriae during the stationary phase of the bacterial population growth cycle and upon induction of various stress conditions. C-di-GMP positively contributes to the expression of CsgD, the key biofilm regulator, activating the expression of the curli regulon (csgBAC), with CsgB and CsgA proteins constituting the curli fiber [9, 35]. As shown in Fig. 1b, the synthesis and degradation of c-di-GMP within this network is maintained by two diguanylate cyclase (DGC)/phosphodiesterase (PDE) pairs: YegE/YhjH (module I) and YdaM/YciR (module II).

While DGC-type enzymes synthesise c-di-GMP from two GTP substrates, PDE-type enzymes are responsible for the degradation of c-di-GMP. As shown in Fig. 1b, besides the degradation function of c-di-GMP, YciR exhibits a second activity: the inhibition of YdaM and MlrA. Importantly, YdaM is the key activator of the transcription factor MlrA, which directly activates transcription at the *csgD* promoter region. A sufficiently high amount of c-di-GMP prevents YciR from inhibiting YdaM, since YciR binds c-di-GMP and starts to degrade it, acting as a trigger enzyme [7]. In turn, unbound (active) YdaM activates MlrA and induces the signalling cascade leading to the expression of the curli operon *csgBAC*.

Previously, the activity of the *csgB* promoter was measured in all possible single, double, triple and quadruple knockout strains of the set of genes *yegE*, *yhjH*, *ydaM* and *yciR* [7]. As depicted in Fig. 1a, despite 15 different genetic backgrounds, only four main expression levels of the *csgB* gene were observed. *E. coli* strains containing a *ydaM*-knockout exhibit a completely absent expression of the curli gene *csgB* (hyperrepressed) or a very low (basal) level expression, as compared to wild type (Fig. 1a). Furthermore, strains containing a *yciR*-knockout reveal an increased (hyperactivated) expression of *csgB* if *ydaM* is present. This knockout background is “blind” with respect to knockouts of module I genes (*yegE* and *yhjH*). Genetic and biochemical analyses allowed Lindenberg *et al.* [7] to derive a model of the regulatory network, which is depicted in Fig. 1b. In this model different functional states are assigned to YciR (I/II) and YdaM (active/inactive).

In order to find evidence for the bistability of the curli signalling system we assigned each of the four different expression levels observed in the genetic knockout experiments at population level (Fig. 1a) a certain single-cell phenotype. To this end, we used the complementary single-cell measurements of the wild type expression of the CsgB protein in Serra *et al.* [11]. These single-cell data suggest that the intermediate expression level of the *csgB* gene at population level corresponds to a heterogeneous mix of curli-on and curli-off cells in the bacterial population. This indicates that the basal and the hyperrepressed level of *csgB* reflects a mixture of cellular phenotypes where the fraction of curli-on cells is significantly lower as compared to the wild type, or it becomes undetectable. In contrast, the hyperactivated phenotype may be due to two different types of single-cell expression levels. Either all cells in the population are in the curli-on mode, or there is a subset of cells in the curli-off mode but their frequency is significantly lower than the frequency of curli-off cells in the wild type population.

### Derivation of the logical model

In order to set up the logical model of the curli regulation system, we described the involved components in terms of logical variables whose values were interpreted as functional states of the components, e.g. active vs. inactive states. For deriving the model we applied a time-scale separation. Thus we assumed that the total levels of involved proteins do not significantly change in contrast to their activity states. We based our assumption on results indicating that protein transcription and translation are significantly slower than post-translational interaction dynamics [36].

Having described the components by means of logical states, logical rules were added to characterise the principles of mutual regulation by the components. In particular, all activating or inhibitory effects indicated by experimental data were incorporated, resulting in the logical model depicted in Fig. 1c. Most of the components were assigned two states (*on* or *off*) corresponding to the situation where the component has either an effect on the system or not. An exception was made for the protein YciR, which was assigned a knockout state (*off*-state) and two different observable activities that are mutually exclusive (PDE activity and YdaM/MlrA-inhibition activity) [7]. Note that YdaM has also two different activity levels. However, since one of them does not have any observable effect, we identified it with the *off*-state.

In addition to the logical variables, we described the semantics of the model via the intertwined regulatory effects. Thus, a regulation is effective if the respective regulator is *on*. In the case of YciR we distinguished between alternative regulations depending on their corresponding effect. A regulation is effective only when YciR occurs in the respective state, including the positive and negative effects (Fig. 1c). Most of the included regulations correspond to the experimentally derived network topology in Fig. 1b, with a few exceptions. The first difference is given by the negative feedback induced by the allosteric product inhibition of c-di-GMP on the catalytic activity of YegE. When molecular numbers are considered, product inhibition (PI) gives rise to an upper limit on the synthesis rate and thus contributes to setting up a homeostatic steady-state level of c-di-GMP [37]. However the semantics of this negative feedback loop do not transfer to the discrete logical model i.e. it is not possible for c-di-GMP to completely inhibit YegE. Since in the logical model, only one discrete value is used as an abstraction for the concentrations of c-di-GMP that are considered as the *on*-state, this negative feedback inhibition has no observable logical effect. Therefore we eliminated the corresponding edge from the logical interaction graph (see Fig. 1c).

Furthermore, the DGC/PDE-pair YhjH and YegE constitute the inputs of the network, which means that they maintain the values that they were initially set to. Finally, we used the system property that the expression of *csgB* is induced if its transcription factor CsgD is expressed. This is the case if MlrA is *on*, since it is in turn the functional activator of *csgD* transcription (Fig. 1b). Therefore, in order to reduce the model, we removed *csgD* and used the boolean component MlrA as the output component of the network. Thus, the *on*-state of MlrA represents the induction of curli expression in the logicalmodel.

At this point we incorporated a sufficient amount of information into the model for deriving all regulatory functions except the functions of c-di-GMP and MlrA. These two components are influenced by multiple regulators, giving rise to a set of alternative logical functions describing their effect on c-di-GMP or MlrA (see Additional file 1 for details). After resolving the constraints derived from the interaction effects, we identified 114 possible regulatory functions for c-di-GMP and 2 for MlrA. Finally, by generating all possible combinations of the functions of the two components, we obtained 228 alternative models for the whole network.

### Formalisation of the experimental data

After specifying all 228 possible alternative logical models (see Additional file 1), we assessed for each model whether it fulfils all constraints given by the observations of the 15 genetic knockout experiments (depicted in Fig. 1a). We evaluated the dynamics of the logical models by employing the so-called asynchronous update that allows for non-deterministic behaviour. It has been previously shown to generate biologically realistic trajectories and has a clear relation to continuous modelling approaches [38]. We modelled a genetic knockout by forcing the respective component to remain in the *off*-state for the whole course of the simulation. Our main focus was on stabilising behaviour, thus we assessed the reachability of stable states with a particular model configuration. A system state is considered as stable if it is not possible to leave it during simulation. As shown in Fig. 1a, there are four distinctive phenotypes, each of which we addressed individually:

- The intermediate (bistable) phenotype was related to the ability to reach a stable state with MlrA *on* and a stable state with MlrA *off* in the logical modelling framework.
- In the hyperrepressed phenotype no curli is being expressed, therefore we required that the stable state with MlrA *off* is reachable, while the *on*-state is not.
- The hyperactivated phenotype exhibits strong expression of curli and therefore we stated that the stable state with MlrA *on* must be reachable. However, unlike the hyperrepressed phenotype, we did not prohibit the inactive stable state, since we could not rule out that this phenotype is generated by a mix of curli-on and curli-off cells.
- Lastly, the basal phenotype exhibits only little, but still measurable expression of curli. However, very weak expression of curli was also observed in the hyperrepressed phenotype (see Fig. 1a). It would therefore be possible to identify the basal with the hyperrepressed phenotype, but this constraint could be spurious. We have therefore decided to not include the basal phenotype in the main analysis.

However, we also tested the validity of the resulting constraint in a second, less conservative analysis step. Here, we assumed that the basal phenotype is equivalent to the hyperrepressed phenotype, see Additional file 1 for details. Since the measurements were conducted in the early stationary phase of the growth cycle (see Fig. 1a), we expected the input components YegE and YhjH to be *on* unless knocked out. Using the stability constraints resulting from experimental data (the knockout data), we formulated 15 properties (bold indices in Table S1 in Additional file 1) that a valid model should fulfil (using the conservative analysis step) and 32 properties using the less conservative analysis step (all rows in Table S1 in Additional file 1). We used these properties to test the entire set of alternative logical models. Subsequently, we identified a valid reduced model topology, as well as parameter constraints that were passed on to the continuous modelling step, as exemplified in Additional file 1 and shown in the “Results” section.

### Derivation of the continuous reaction-rate model

Based on the results from the logical modelling step (see Additional file 1), we included three system components as dynamical variables into the continuous model: the amount of c-di-GMP (*x*_1_), the amount of YciR molecules in its role as a YdaM inhibitor (*x*_2_) (i.e. YciR II in Fig. 1b) and the amount of active YdaM molecules (*x*_3_). Due to the time-scale separation assumption, transcription and translation were assumed to be negligible and thus the total amount of YciR and YdaM was assumed to be constant (parameters *YciRtot* and *YdaMtot*, respectively). Two further system components, YegE and YhjH, were included as parts of the maximum catalytic velocity parameters (*V*_max1_ and *V*_max2_, respectively). We did not include MlrA into the model since its activity reflects the activity of YdaM. This was supported by the results of the logical modelling step indicating that MlrA does not influence the interactions between module I and module II proteins (we assumed that YdaM and MlrA do not compete for YciR, as discussed later). Details of the derivation of reaction rates of the time-continuous model are given in the Additional file 2.

According to the network wiring validated in the logical model checking step (see “Results”), the final continuous model was composed of interactions given either by the production or degradation reactions of c-di-GMP. Based on the elementary catalytic reactions, we derived Michaelis-Menten rates by applying the quasi-steady-state assumption (see Additional file 2 for details and derivations).

C-di-GMP synthesis by YegE and YdaM respectively occurs with rates *V*_1_ and *V*_2_, while c-di-GMP degradation by YhjH and YciR are modelled by rates *V*_2_ and *V*_3_, respectively. The remaining four rates (*V*_5_, *V*_6_, *V*_7_ and *V*_8_) relate to the reversible transitions between different functional states of YciR and YdaM, as outlined in Additional file 2 and summarised in Table 1.

**Table 1:** Biochemical rates of molecular interactions involved in the curli regulation network. Reaction rates were derived according to catalytic properties of c-di-GMP regulation and protein-protein interactions identified in [7]. Since the focus was on post-translational dynamics, the protein expression levels of all DGCs and PDEs were held constant using the parameters *V*_max1_ (YegE), *V*_max2_ (YhjH), *YdaMtot* (YdaM) and *YciRtot* (YciR)

Rate function	Description of the reaction
$V_{1} = \frac {V_{\max 1}}{1 + x_{1}/K_{\mathrm {i}}^{\text {YegE}}}$	Synthesis of c-di-GMP by YegE
$V_{2} = \frac {V_{\max 2} x_{1}}{x_{1} + K_{\mathrm {m}}^{\text {YhjH}}}$	Degradation of c-di-GMP by YhjH
$V_{3} =(\textit {YciRtot} -x_{2})\frac {k_{\text {YciRact}} x_{1}}{x_{1} + K_{\mathrm {m}}^{\text {YciR}}}$	Degradation of c-di-GMP by YciR
$V_{4} = \frac {k_{\text {YdaMact}} {x_{3}^{n}}}{\left (K_{\mathrm {d}_{\text {polymer}}}^{\text {YdaM}} \right)^{n} + {x_{3}^{n}}}$	Synthesis of c-di-GMP by YdaM (no PI)
$V_{5} = k_{\text {YciRde}} x_{2} \frac {x_{1}}{x_{1} + K_{\mathrm {d}}^{\text {YciR}}}$	Transition from YciR II (YdaM inhibiting)
	to YciR I (PDE activity) due to c-di-GMP
	binding
*V*_6_=*c*_6_(*Y* *c* *i* *R* *t* *o* *t*−*x*_2_)	Transition YciR I → YciR II
$V_{7} = k_{\text {YdaMde}} x_{3} \frac {x_{2}}{x_{2} + K_{\mathrm {d}}^{\text {YdaM}}}$	Inhibition of YdaM due to binding of YciR II
*V*_8_=*c*_8_(*YdaMtot*−*x*_3_)	Re-activation of YdaM

Based on the reaction rates, we derived an ODE model describing the interaction dynamics of module I and module II proteins and the signal transduction via the regulation of c-di-GMP levels in the cell (Eq. 1). This results in the following system of ordinary differential equations (ODEs):

(1) $$\begin{array}{@{}rcl@{}} \frac{d}{dt} x_{1} &=& V_{1} + V_{4} - \left(V_{2} + V_{3} \right),  \\ \frac{d}{dt} x_{2} &=& -V_{5} + V_{6},  \\ \frac{d}{dt} x_{3} &=& -V_{7} + V_{8},  \end{array} $$

where the system variables *x*_1_, *x*_2_, and *x*_3_ denote the molecular concentrations of c-di-GMP, YciR in the YdaM-inhibition state and active YdaM, respectively. The reaction rate functions are stated in Table 1.

Setting the second equation of the ODE system (1) to zero, we obtained the steady state equation for the amount of YciR molecules in both activity states (YdaM/MlrA-inhibition activity state *x*_2_ and PDE activity state *YciRtot*−*x*_2_):

(2) $$\begin{array}{@{}rcl@{}} x_{2}^{\text{ss}} &=& \frac{c_{6} \textit{YciRtot}}{c_{6} + k_{\text{YciRde}} \frac{x_{1}}{x_{1} + K_{\text{d}}^{\text{YciR}}}} = \frac{K_{\text{d}}^{\text{YciRact}} \textit{YciRtot}}{K_{\text{d}}^{\text{YciRact}} + \frac{x_{1}}{x_{1} + K_{\text{d}}^{\text{YciR}}}},  \end{array} $$

where we introduced the equilibrium binding constant given by

$$K_{\text{d}}^{\text{YciRact}} = \frac{c_{6}}{k_{\text{YciRde}}}. $$

Furthermore, by setting the third equation of the ODE system (1) to zero and substituting *x*_2_ with $x_{2}^{\text {ss}}$, we obtained the equation for the amount of active (*x*_3_) and inactive (*YdaMtot*−*x*_3_) YdaM molecules at steady state:

(3) $$\begin{array}{@{}rcl@{}} x_{3}^{\text{ss}} &=& \frac{c_{8} \textit{YdaMtot}}{c_{8} + k_{\text{YdaMde}} \frac{x_{2}^{\text{ss}}}{x_{2}^{\text{ss}} + K_{\mathrm{d}}^{\text{YdaM}}}} = \frac{K_{\mathrm{d}}^{\text{YdaMact}} \mathit{YdaMtot}}{K_{\mathrm{d}}^{\text{YdaMact}} + \frac{x_{2}^{\text{ss}}}{x_{2}^{\text{ss}} + K_{\mathrm{d}}^{\text{YdaM}}}},  \end{array} $$

where

$$K_{\text{d}}^{\text{YdaMact}} = \frac{c_{8}}{k_{\text{YdaMde}}}. $$

Substituting *x*_2_ and *x*_3_ with $x_{2}^{\text {ss}}$ and $x_{3}^{\text {ss}}$ in the first equation of the ODE system (1) yields a one-dimensional ODE. Its roots indicate the levels of c-di-GMP $x_{1}^{\text {ss}}$, which, in conjunction with the steady state Eqs. 2 and (3), give rise to the fixed points $\mathbf {x}^{\text {ss}} = \left \{x_{1}^{\text {ss}},x_{2}^{\text {ss}}, x_{3}^{\text {ss}}\right \}$ of the entire ODE system. A fixed point *x*^ss^ is a stable steady state if all eigenvalues of the Jacobian matrix of the ODE system (1) at *x*^ss^ are real and negative.

### Parameter identification

We estimated the parameters of the ODE model (1) by using a rejection sampling scheme. In the first step we sampled random parameter values using a uniform proposal distribution with bounds previously described in the literature or using physiologically meaningful ranges, as shown in Table 2 and indicated by grey background shading in Additional file 4: Figure S2. Note that for the ODE system (1) the identified parameters with units *molecules* were scaled by the system volume *Ω* that was set to *Ω*=1*μ*m^3^ [39].

**Table 2:** Ranges of the uniform proposal distribution used for parameter sampling. Kinetic rate parameters were sampled using a uniform proposal distribution with ranges as depicted in the table. Physiologically meaningful boundaries were chosen if no specific information was available from previous studies (last three rows). In the case where the parameter distribution was post-hoc trimmed, the effective range is shown and the original sampling range is given in brackets

Parameter names	Unit	Lower bound	Upper bound	References
$K_{\text {m}}^{\text {YhjH}}$	molecules	100	3000	[35, 49]
$K_{\text {m}}^{\text {YciR}}$	molecules	100	1000 (3000)	[50] p. 103, [35]
$K_{\text {d}}^{\text {YciR}}$	molecules	100	3000	[50] p. 103, [35]
$K_{\text {i}}^{\text {YegE}}$	molecules	0	2000	[37]
*V*_max1_, *V*_max2_, *k*_YdaMact_, *k*_YciRact_	molecules /*s*	0	2000	[49, 51]
$K_{\text {d}}^{\text {YdaM}}$	molecules	100	3000	physiol. range
*YciRtot*, *YdaMtot*, $K_{\text {d}_{\text {polymer}}}^{\text {YdaM}} $	molecules	0	2000	physiol. range
*k*_YciRde_, *k*_YdaMde_	molecules /*s*	0	2000	physiol. range

In order to reduce the parameter search space, we applied a set of constraints. Firstly, we required that the proposal values of the parameters *YciRtot* and *YdaMtot* are equal, as suggested by protein assays performed by Lindenberg *et al.* [7]. Furthermore, we included a constraint resulting from the logical modelling step requiring that the catalytic activity of YciR is weaker than the catalytic activity of YdaM, i.e. *k*_YciRact_≤*k*_YdaMact_ (see Additional file 1 for derivation).

A parameter proposal was accepted if the following set of qualitative requirements was fulfilled:

- the wild type model and the double knockout *yegE*/*yhjH* exhibit two stable states,
- the single-gene knockout mutant lacking *yhjH* either exhibits one or two stable states,
- the single-gene knockout mutant lacking *yegE* exhibits one stable state.

Note, that the remaining knockout data in Fig. 1a were not included for kinetic parameter sampling since they contain genetic knockouts of *ydaM* or *yciR*. Setting the amount of each of these two components to zero disrupts the structure of the ODE (1) since these two components are described by system variables (as opposed to the activities of YegE and YhjH, described by model parameters). The parameters were estimated using a fixed Hill constant *n*=4. This is supported by the tendency of YdaM to build tetramers *in-vitro* [7], in line with previous work suggesting multimerisation as a possible source for ultrasensitivity [40].

In the second step we conducted a rejection sampling with a multivariate Gaussian proposal distribution by accepting the sampled parameter sets if all stability constraints from the first step were fulfilled. The mean *μ* of the proposal distribution was given by the parameter sets identified in the first (uniform) sampling step and the standard deviation *σ* was chosen sufficiently large to cover a large sampling space but minimise the probability of negative sampling proposals (coefficient of variation *σ*/*μ*=0.25). In the case of the parameter $K_{\text {m}}^{\text {YciR}}$ all sampled parameter sets, where the range of 1000 molecules was exceeded, were post-hoc rejected due to experimental results suggesting this upper bound [41]. Additionally, we post-hoc selected only those parameter sets which fulfilled the validity criterion for the Michaelis-Menten approximation in deterministic and stochastic models (see Additional file 2, Eq. (S12)).

### Chemical Master Equation of the curli regulation system

The stochastic model of curli regulation and the validity of the translation of continuous rates into stochastic propensities are discussed in Additional file 2. In order to solve the corresponding Chemical Master Equation and to compute switching probabilities between the stable states, we generated a sufficiently large amount of sample trajectories using the Stochastic Simulation Algorithm [42]. For computing the probability of stable states (stationary distribution) we used sufficiently large simulation times ensuring the equilibration of the system. For instance, a simulation time of 100s for the parameter set used for generating Fig. 4 was sufficient, as shown in Additional file 5: Figure S3.

We then computed switching probabilities and switching times. A switch was detected if an SSA trajectory that was started in a stable state reached the neighbourhood of the opposite stable state within a maximal simulation time *T*=100s. For instance, if a system trajectory **X** was started in the stable state **X**^ss1^, then a switch to the stable state **X**^ss2^ was detected if

(4) $$\begin{array}{@{}rcl@{}} \sum_{i=1}^{N} X_{i} - X_{i}^{\text{ss}2} \leq \sum_{i=1}^{N} \epsilon \left(\left|X_{i}^{\text{ss}1} - X_{i}^{\text{ss}2}\right|\right)  \end{array} $$

where *ε* is a neighbourhood parameter that we set to 0.1 and *N* denotes the number of system components (3 components in the case of our model). The stable states **X**^ss1^ and **X**^ss2^ were computed as the steady states of the deterministic reaction-rate model (1) and confirmed by identifying the modes of the solution of the CME after a sufficiently long stochastic simulation time.

## Results

We applied logical model checking in order to assess the potential of the experimentally derived wild type interaction network (Fig. 1b) to exhibit bistable dynamics and at the same time to fulfil the stability constraints from the various genetic knockout strains (Fig. 1a).

Following the logical model checking step, we then incorporated currently available information on biochemical reactions in order to derive a more detailed reaction-rate model of curli regulation.

### Logical analysis: topology and parameters

As a first analysis step, we tested the consistency of the candidate models, constructed in the “Methods” section, with the 15 constraints (Table S1 in Additional file 1, rows indicated with a bold index) derived from the experimental observations (Fig. 1a). As a result, we found that 10 out of 228 possible models were in agreement with all constraints (see Table S2 in Additional file 1). Using a comparative analysis of this set of 10 feasible models we obtained a number of results yielding meaningful insights into signalling mechanisms of curli regulation and supporting subsequent continuous modelling. In the following, we first describe the essential features of the system resulting from common properties of the feasible models and afterwards we present the consequences for the continuous modelling step.

**Fig. 2:**
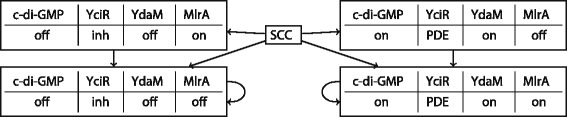
Simulation trace of the system where both inputs YegE and YhjH are set to active states. Values of the remaining four nodes create 2^4^=16 different combinations, 12 of which form the strongly connected component (SCC) and the bottom two are the stable states, indicated by the self-loops. The depicted simulation trace is shared among all final models

In Fig. 2 we illustrate the dynamical behaviour that is shared by all 10 models. As ensured by the constraints, all models support bistability given that both inputs (YegE and YhjH) are active. This means that the model behaviour is non-deterministic and can asymptotically end in either one of the two stable states distinguished by the presence of MlrA. Interestingly, for all models these stable states do not only coincide in that they represent *expression* or *no expression* of curli, but also in the activity of all other model components. That is, we can completely characterise the two possible equilibrium states when the input components are *on*. Furthermore, an inspection of the regulatory functions of the 10 remaining models indicates that there are five possible regulation mechanisms for c-di-GMP, which exhibit noteworthy commonalities. The most prominent pattern is that c-di-GMP is in most cases adopting the value of YdaM, whereas the value of YciR is almost uncorrelated with the update of c-di-GMP. This observation allowed us to identify YdaM as the most influential regulator of c-di-GMP. For a more detailed discussion and an explicit listing of the regulatory functions see Additional file 1.

Apart from yielding insights into the modelled system, the logical approach could also be used to validate crucial assumptions underlying the continuous reaction-rate model: Firstly and most importantly, we validated the network structure underlying the continuous reaction-rate model. To this end, as described above, we identified a non-empty set of logical models fulfilling the experimental constraints and exhibiting bistability. Furthermore, by inspecting the set of 10 feasible models, we observed that despite of two different choices for the regulation of MlrA, the realisation of the core circuit YdaM—YciR—c-di-GMP is independent of the choice of the regulatory function of MlrA. Furthermore, MlrA is an output component and thus does not influence any other components in the network, allowing for model reduction by completely removing MlrA in the continuous modelling step. This gave rise to a core model that generates bistability, containing only c-di-GMP, YdaM and YciR as dynamic variables.

In a second step, we asked if, besides the already incorporated (conservative) constraints, the network topology also fulfils the constraints related to the less conservative interpretation of the basal expression phenotype, as e.g. observed in the mutant strain with a *yegE*-knockout (Fig. 1a, see “Methods” for a description of model constraints). To this end, we made the assumption that the basal phenotype is equivalent with the hyperrepressed phenotype. This assumption is partly supported by the knockout data, which suggest that these two phenotypes are indeed equivalent w.r.t. to the core network (YdaM, YciR and c-di-GMP) and only differ by the basal YdaM-independent activity of MlrA inhibited by the activity of YciR (e.g. compare *csgB* expression in the mutant strain lacking *ydaM* and the double-knockout mutant strain lacking *ydaM* and *yciR*). Overall we generated two constraints from each knockout experiment in Fig. 1a, giving rise to 32 constraints (Additional file 1: Table S1). As a result, we identified one valid model fulfilling *all* constraints (including the less conservative constraints obtained by identifying the basal- and hyperrepressed phenotype, see first row in Table S2, Additional file 1).

Finally, we were able to translate our findings on the regulation of c-di-GMP into a parameter constraint for the ODE model. Our result stating that the activating effect of YdaM overcomes the inhibitory influence of YciR under certain conditions yields the kinetic parameter inequality *k*_YciRact_<*k*_YdaMact_ in the continuous setting (see Additional file 1 for details).

In summary, we were able to transfer four results into the continuous modelling step. Firstly, the network structure postulated in Fig. 1c, is a valid basis for a continuous reaction-rate model of curli regulation and supports the experimentally derived network topology. Secondly, the core model giving rise to bistable behaviour is based on the interaction between YdaM, YciR and c-di-GMP (with YegE and YhjH as model inputs). Thirdly, we have shown that the network topology also fulfils the constraints resulting from the equivalence assumption of the basal and hyperrepressed phenotypes. Thus, this assumption can be used as a constraint in the continuous modelling step. Finally, we obtained a parameter constraint for the catalytic activity of YciR in comparison with the activity of DGC enzyme YdaM.

### Reaction-rate model of interaction between the DGC/PDE modules

In the subsequent step, we analysed the dynamics of the system after translating the logical network into a continuous reaction rate model. We then determined the stable states of the system (see “Methods”). Since in the logical modelling step the activity of MlrA was shown to almost completely reflect the activity of YdaM, we only focused on interactions of the module I and module II proteins, upstream of MlrA, as the regulatory core of the network (Fig. 1d and “Methods”). Note, that this model reduction is valid under the assumption that MlrA and YdaM can simultaneously bind YciR i.e. there is no competition (see “Discussion”). We used the bistability of the wild type strain and the stability properties of the single-gene knockout strains lacking either *yegE* or *yhjH* (Fig. 1a) as acceptance criteria for parameter inference (see “Methods”).

**Fig. 3:**
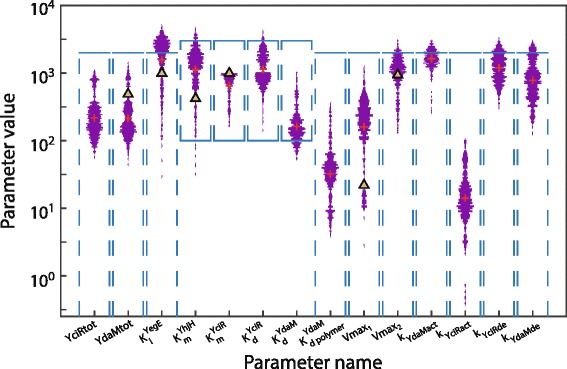
Distribution of sampled parameters along with reference parameter values from the literature. Parameter distributions were obtained using rejection sampling where, if possible, the bounds of the proposal distribution were obtained from the literature (see Table 2) and are outlined by blue boxes. In addition, the empirical means of each parameter are shown (red crosses). The first eight parameters on the x-axis have units *molecules*, the following six parameters have units molecules /*s*. The individual values of each parameter set are provided in the Additional file 3. For a comparison, experimentally measured values of similar parameters are depicted, if available (triangles). Following parameter pairs are indicated for comparison (model parameter/parameter from literature): 1. *YdaMtot*/Amount of DgcA in *E. coli* ≈490 molecules, obtained from [37] and normalised by the dry weight of the cell obtained from the Bionumbers database, BNID 100009 [52]. 2. $K_{i}^{\text {YegE}}$/ *K*_*i*_ of DgcA ≈1000 molecules, obtained from [37] and normalised by the volume of *E. coli* cells. 3. $K_{m}^{\text {YhjH}}$/ *K*_*m*_ of the PDE-enzyme CC3396 from *Caulobacter crescentus* ≈420 molecules, obtained from [49] 4. $K_{m}^{\text {YciR}}$/estimated maximal *K*_*m*_ of YciR ≈1000 molecules, obtained from [50], page. 103 and normalised by the volume of *E. coli* cells. 5. *V*_max1_ of YegE/ *V*_max_ of DgcA ≈22 *s* ^−1^ obtained from [37] and normalised by the amount of DgcA proteins and by the dry weight of the cell. 6. *V*_max2_ of YhjH/ *V*_max_ of the PDE-enzyme CC3396 from Caulobacter crescentus ≈937 *s* ^−1^ obtained from [49] and normalised by the dry weight of the cell. Note that the Hill parameter was fixed at *n*=4 (see text for explanation) and the parameters *c*_6_ and *c*_8_ were fixed at the value 10 *s* ^−1^ due to structural non-identifiability w.r.t parameters *k*_YciRde_ and *k*_YdaMde_, respectively

Using 2·10^6^ parameter proposals, our sampling procedure yielded 608 feasible parameter sets depicted in Fig. 3 (log-scale) along with experimentally determined parameters in related systems. The individual parameter values are listed in the Additional file 3. Note also Additional file 4: Figure S2 showing the parameter distribution on a linear scale along with the proposal distribution.

### Bifurcation analysis and comparison to genetic knockout experiments

Previously, it was shown that protein-protein interactions between the module II proteins YciR and YdaM induce a switching point in this system requiring a sufficient amount of c-di-GMP in order to relieve the inhibition of YdaM by YciR and enable downstream signal transduction [7]. In the following, we analysed the potential role of the regulatory parameters of c-di-GMP in generating bistability. Since experimental results suggest that YdaM is the main component transmitting the signal further downstream to MlrA, we used the amount of active YdaM molecules as the read-out parameter of our analysis. Note that the stable states with a high amount of YdaM molecules are characterised by a high level of c-di-GMP and a low level of YciR II and vice versa, in line with the stable states of the logical model (Fig. 2). By construction of the rejection sampling, all identified parameter sets shared equivalent stability properties of the wild type and genetic knockout strains. Therefore, in order to analyse the dependence of the stable states on model parameters we selected a representative parameter set from the sampled distribution (see Additional file 3, parameter set 1 listed in the first data row of the table).

**Fig. 4:**
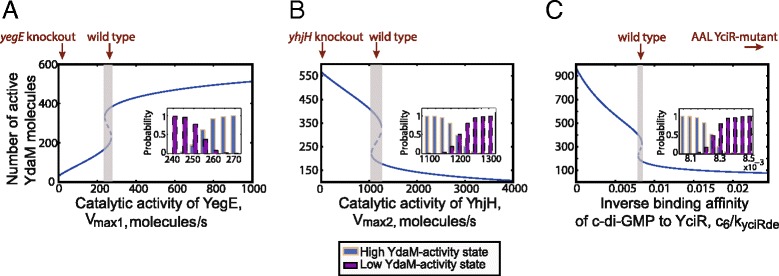
Bifurcation analysis of key system parameters *V*_max1_, *V*_max2_ and the complex parameter *c*_6_/*k*_YciRde_. The stable states are shown by continuous lines while unstable fixed points are indicated by dashed lines. Note that each stable level of active YdaM is characterised by a distinct stable amount of c-di-GMP and YciR II (not shown). The parameter regions inducing bistability are marked by grey areas. Inlay plots show stochastic simulations of the system within the bistable region, indicating the probability (relative amount of cells) to contain a *high* or *low* amount of active YdaM molecules. The initial condition for the stochastic simulations was set to *x*_1_=100,*x*_2_=0,*x*_3_=768 and 10^3^ simulations were conducted for each parameter combination of the bifurcation analysis. A sufficiently large simulation time of 100 seconds was chosen in order to ensure equilibration. In each experiment A-C all the other parameters were kept fixed corresponding to the parameter set 1 in Additional file 3 (first data row of the table)

The parameters *V*_max1_ and *V*_max2_ are composed of the product of catalytic activity and the amount of the DGC-type enzyme YegE and the PDE-type enzyme YhjH, respectively. We varied these two parameters in order to analyse the impact of the amount and activity of this DGC/PDE pair on signal transduction. For each of the two parameters we identified three different types of qualitative dynamics. At values of *V*_max1_ slightly below 238 *molecules* /*s* the system exhibits monostable dynamics (Fig. 4a). A further decrease of this parameter reduces the amount of active YdaM proteins at steady state eventually hitting the minimal level of active YdaM proteins when YegE activity is absent (*V*_max1_=0). This is supported by experimental measurements of *csgB* expression in *yegE* knockout mutants exhibiting basal YdaM-independent level of *csgB* expression (Fig. 1a) [7, 8]. With an increasing *V*_max1_, the amount of active YdaM molecules increases as well, until the system becomes bistable with two possible stable levels of active YdaM molecules in a region between 238 and 272 *molecules* /*s*. This type of qualitative dynamics agrees with the phenotypic heterogeneity of *csgB* expression observed in the wild type strain [10, 11]. The stochastic simulations of the model indicate that within this parameter region the cells divide into two subpopulations with different levels of active YdaM molecules (Fig. 4a, inlay plot). Finally, a further increase of *V*_max1_ makes the system monostable again with a high level of active YdaM molecules (corresponding to the curli-on state). A similar analysis of the YhjH-activity parameter *V*_max2_ in the second experiment exhibited the contrary bifurcation behaviour, where the system becomes monostable with a high level of active YdaM molecules as *V*_max2_ decreases (Fig. 4b). This corresponds to the hyperactivated expression of *csgB*, experimentally measured in *yhjH*-knockout mutants (Fig. 1a).

In the third experiment we varied the complex parameter *c*_6_/*k*_YciRde_. This parameter is inversely proportional to the binding affinity of c-di-GMP to YciR and it has an opposite bifurcation behaviour as compared to *V*_max1_. Values of *c*_6_/*k*_YciRde_ below 7.92·10^−3^, i.e. a high affinity of c-di-GMP to YciR, give rise to a single steady state with a high level of active YdaM molecules. Between 7.92·10^−3^ and 8.39·10^−3^ the system becomes bistable with two different levels of active YdaM molecules, which corresponds to the wild type dynamics. The corresponding stochastic simulations indicate that within this parameter region the probability of the system is divided between the two stable states, which is in agreement with the bistable dynamics of *csgB* expression. Finally, at values higher than 8.39·10^−3^, i.e. a further decrease of the binding affinity of c-di-GMP to YciR, the system becomes monostable again, exhibiting low level of active YdaM molecules (curli-off). This is supported by experimental data indicating a basal expression of *csgB* in mutant strains where the EAL domain was mutated to an AAL domain, thus diminishing the binding affinity of c-di-GMP to YciR [7]. The model suggests that the observed basal expression of curli might be a result of a single steady state with a low level of active YdaM proteins. Note that despite individual differences in the exact parameter values *V*_max1_, *V*_max2_ and *c*_6_/*k*_YciRde_ between distinct feasible parameter sets at bifurcation points, the qualitative dynamics described above can be generalised to all identified parameter sets shown in Fig. 3.

### Dynamics of stochastic switching between different activity levels of YdaM

Although the identified parameter sets are qualitatively equivalent with respect to their stability properties (i.e. number of stable states in the wild type, *yegE*- and *yhjH*-knockout mutant strains and bifurcation behaviour), the equilibrium *probabilities* of each stable state and the *dynamics* of switching between the stable states might differ significantly. Potential spontaneous switching between the stable states is an important property of stochastic multistable systems. By generating sample trajectories of the stochastic model one can show that on very short time scales the probability of each stable state is fully determined by the initial level of the key components in the system, while after sufficiently long simulation times(∼100s) the switching dynamics between the stable states marginalises the effect of initial molecular levels by inducing equilibration (see Additional file 5: Figure S3).

**Fig. 5:**
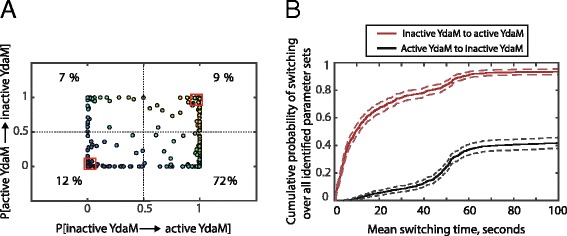
Classification of identified parameter sets according to their mean switching times and switching probabilities between the two stable states. **a**. Each of the feasible parameter sets is plotted according to the switching probability between the two stable states, given a maximal simulation time of 100 seconds. The parameter sets are classified into 4 regions which are divided by lines indicating a probability $\mathcal {P}=0.5$ of a switch in either direction. The colours represent the probabilities of switching in both directions, ranging from blue to yellow (low and high probability of switching in either direction, respectively). The red boxes in the lower left and upper right corners indicate parameter regions inducing a low probability ($\mathcal {P} \leq 0.1$) and high probability ($\mathcal {P} \geq 0.9$) of switching in either direction, respectively. **b**. Cumulative distribution of the switching times over all feasible parameter sets. For each of the 608 identified parameter sets 10^3^ SSA simulations were performed by starting the system in the stable state either corresponding to the curli-off or curli-on state i.e. low/high amount of c-di-GMP, high/low amount of YciR in the YdaM/MlrA-inhibition state and low/high amount of active YdaM molecules, respectively. Each simulation was stopped if a switch to the opposite stable state was observed or if the maximal simulation time of 100 seconds was reached (see Eq. (4) for the definition of a switch). The mean switching time was computed over those trajectories that exhibited a switch for a given parameter set

In the following, we analysed the switching behaviour between the two stable states (low vs. high level of active YdaM molecules) using all identified parameter sets. For each parameter set, we computed the probability of switching within a simulation time of 100 seconds. The result for each of the 608 identified parameter sets with regard to switching between the low and high YdaM activity is shown in Fig. 5a (each parameter set is represented by a circle with the two switching probabilities as its coordinates). A separation of the parameter space into four regions according to the switching probabilities between the two stable states indicates a certain asymmetry of the switching dynamics between the two states. For most of the identified parameter sets (72 *%*) we observed a high probability of switching ($\mathcal {P}\geq 0.5$) from the low YdaM activity state to the high activity state while exhibiting a low inverse switching probability ($\mathcal {P}<0.5$). These parameters induce an alert-type behaviour, where the system is very likely to switch to the curli expression state and most probably will remain in this state for some time. The number of these parameter sets exceeded the number of parameter sets exhibiting the opposite behaviour by an order of magnitude i.e. only 7 *%* of parameters indicated a small probability of switching from low YdaM activity level (curli-off) to a high YdaM activity level (curli-on), but a high probability of switching in the opposite direction. Finally, a significant amount of feasible parameters sets exhibited very low switching probability in either direction (12 *%*), representing a possible robust differentiation of the bacterial population into two phenotypes, while (9 *%*) indicated high switching probabilities in both directions.

In addition, we computed the time to switch between the two stable states. A summary plot for all simulations (all parameter sets) is shown in Fig. 5b, indicating that the time required to switch from the low to the high level of active YdaM (switch from curli-off to curli-on) was significantly shorter than for the reverse direction. When starting from the curli-off (low YdaM activity) state, a switch was observed in 93 *%* of all model parameterisations within 100 seconds. However, only slightly over 41 *%* exhibited a switch from the high YdaM activity state to the low YdaM activity state after this period (Fig. 5b). This difference in the times for switching in different directions is in line with the overrepresentation of parameter sets with a high probability of switching from the low to the high YdaM activity state and low probability of switching in the reverse direction, as discussed above (Fig. 5a).

**Fig. 6:**
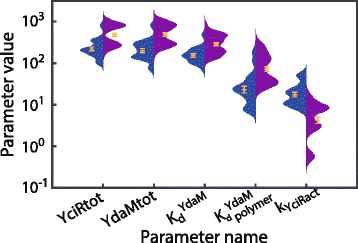
Comparison of distributions of parameter sets inducing frequent switching to parameter sets with rare switching. Stochastic simulations were performed as described in Fig. 5. The distributions of two parameter sets were compared: those with a high probability of switching $\mathcal {P}\geq 0.9$ in both directions (blue, 21 parameter sets) and those with a low probability of switching $\mathcal {P}\leq 0.1$ (red, 77 parameter sets), as indicated by red boxes in Fig. 5 a. A Wilcoxon ranksum test was applied for detecting significant differences at a significance level *p*≤0.001. The parameters exhibiting no significant differences between the two sets are not shown

In order to identify the factors that give rise to the differences in switching behaviours, we compared the corresponding parameter sets. We identified significant differences between the distributions of several parameters inducing frequent switching in both directions ($\mathcal {P}\geq 0.9$, 21 parameter sets) and those inducing very rare switching in both directions ($\mathcal {P} \leq 0.1$, 38 parameter sets) i.e. the parameter sets marked by the two red boxes in Fig. 5a. As shown in Fig. 6, there are significant differences (Wilcoxon ranksum test, *p*<0.001) between the two parameter sets in terms of the total levels and/or activities of the PDE-type enzyme YciR (*YciRtot*, *k*_yciRact_) and the DGC-type enzyme YdaM $\left ({YciRtot}, K_{\text {d}}^{\text {YdaM}} ~~\text {and}~~ K_{\text {d}_{\text {polymer}}}^{\text {YdaM}}\right)$.

This suggests that changes of expression and activity levels of the DGC and PDE enzymes due to e.g. transcriptional regulation might modulate the responsiveness of the system.

**Fig. 7:**
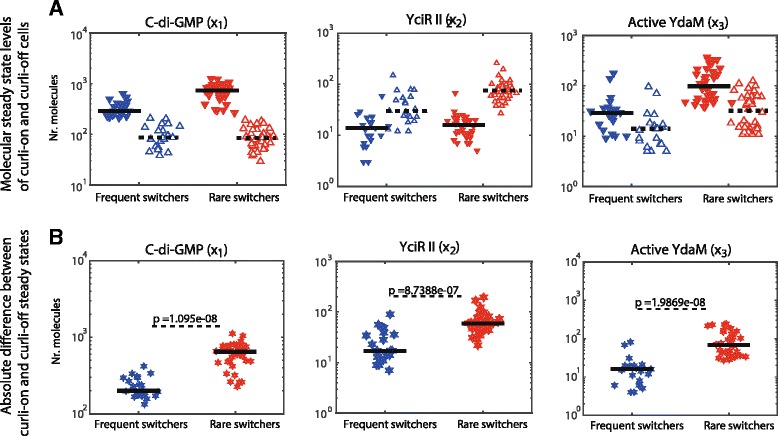
Comparison of steady state levels of key system components within curli-off and curli-on phenotypes depending on switching behaviour. Panel **a** (upper figure row): steady state levels of system components are shown as indicated above each subfigure for parameter sets inducing bistability with frequent switching ($\mathcal {P}\geq 0.9$, blue) and rare switching ($\mathcal {P} \leq 0.1$, red) between curli-off and curli-on states. Within each of these two groups the steady state levels of curli-on and curli-off states are shown (filled, downwards pointing triangles and empty, upwards pointing triangles, respectively). The steady state levels were computed using the ODE system (1) and, for consistency, compared with the modes of the solution of the Chemical Master Equation obtained by SSA sampling (Additional file 5: Figure S3). Panel **b** (lower figure row): the absolute difference between the molecular levels of system components between the curli-on and curli-off states is shown. The difference of molecular levels is computed for parameter sets inducing high and low probability of switching (blue and red hexagrams, respectively) between the curli-on and curli-off states. The *p*-values indicate the significance of the separation between the two distributions (Wilcoxon ranksum test)

We compared the steady state levels of c-di-GMP, YciR II and active YdaM between parameter sets inducing frequent vs. rare switching dynamics between the curli-on and curli-off states *in both directions* (Fig. 7a). This graphic highlights the barrier that the system is required to overcome in order to switch to the opposite steady state. As shown in Fig. 7b the steady state levels in the curli-on and curli-off states are significantly more distant for parameters inducing rare switching than those inducing frequent switching (in both directions), possibly constituting a mechanism for controlling switching dynamics. Furthermore, we compared parameter sets inducing a high switching probability in only one direction but a low probability in the reverse direction (i.e. a comparison of parameters in the upper left corner to the parameters in the lower right corner in Fig. 5a). Significant differences between these data sets with respect to parameter values or steady state levels could not be found.

Overall, besides the potential of the curli regulation system to induce bistable dynamics, our stochastic modelling results indicate alternative feasible scenarios of switching between the stable states. At the one extreme, the cells may be able to rapidly switch their phenotype and at the other extreme they may differentiate into two stable phenotypes. A difference between these two scenarios may be given by distinct expression levels of the involved proteins which regulate the energetic barrier separating the two stable states. It should be noted that certain reactions of the continuous model do not allow to distinguish between the impact of individual parameters as, for instance, the catalytic velocity of c-di-GMP degradation is determined by the product of two parameters: *YciRtot* and *k*_YciRact_. Therefore, additional kinetic data may help in future to further dissect the impact of reaction rate parameters on bistability and switching dynamics of curli expression.

## Discussion

Multistability is a recurring mechanism in biological systems, enabling cellular heterogeneity and differentiation at the phenotypic level [6]. In order to understand the principles of its regulation, a thorough combination of wet-lab experimentation and mathematical modelling is required. Many computational studies of bistable systems focused *either* on the network topology [17, 18] or on reaction kinetics [19, 21]. Here, we combined these two approaches. This enabled us to deal with heterogeneous data sources, such as expression levels in various knockout strains and kinetic rate parameters from *in-vitro* studies.

While multistability in signalling networks may be attributed to transcriptional and translational regulation as well as post-translational dynamics, in this study, we focused on molecular interactions at the post-translational level.

In our modelling pipeline we utilised three modelling frameworks in sequence to fully exploit the available data and investigate different aspects of the system on appropriate levels of abstraction. Insights from each modelling- and analysis step were passed on in the pipeline and constituted, together with additional biological information accessible within the next higher detail-resolving formalism, the foundation for more elaborate models.

In a first step we used a constraint-based logical modelling approach enabling us to extract essential model characteristics needed to reproduce all available experimental observations. More precisely, we identified a set of logical models consistent with the suggested network topology that generates bistability in the wild type strain and fulfils all constraints given by the genetic data. Furthermore, the logical modelling step revealed that the bistable distribution of the molecular pool of *active* YdaM molecules (not bound by YciR) is the key determinant of phenotypic heterogeneity of curli expression observed in single-cell experiments. This insight allowed for a model reduction (compare Fig 1b and d). The implicit assumption made here was that MlrA and YdaM do not compete for binding to YciR, making MlrA a downstream component of the core regulatory network. Structurally, this is justified by the possibility of independent and simultaneous binding of YdaM and MlrA to YciR due to its large interaction domain (EAL-domain) [43] and different binding modes of YdaM and MlrA on that domain. In addition, genetic data from Lindenberg *et al.* [7] yielded functional arguments against competition between YdaM and MlrA. Within our pipeline, the main result from the logical analysis passed on to the higher resolution modelling is the validation of the underlying network structure. We verified that the reduced topology shown in Fig 1d can carry a dynamical model in agreement with all available data.

Beyond purely topological insights, a closer look at the regulatory mechanisms encoded by the logical functions can also be exploited to derive parameter constraints for the continuous reaction-rate model. Analysing the impact of different regulators on a target in different system states may yield information on relations between production and decay rates. Theoretical results in this direction have been shown for specific classes of ODEs, see e.g. [38], but they are not yet widely applicable. We suggested how to exploit this idea in a well-supported case for the curli regulation network and identified its potential for the sampling of kinetic parameters. Still, for a systematic application of this strategy the theoretical groundwork needs to be further extended.

An even closer intertwining of the formalisms would also have been possible. Automatic conversion procedures could have been used to lift a more abstract model into a more resolved formalism, e.g., to derive a generic ODE system from a given logical model as suggested in [44]. However, this approach yields models that cannot incorporate additional mechanistic knowledge (e.g. reaction parameters) in contrast to our approach, where we aimed at constructing a biologically realistic model by utilising all available kinetic information. As a consequence, the continuous model in our approach does not necessarily mimic the dynamics of the coarser models in all system states. It rather provides a complementary view based on the much higher detail resolution that allows to re-evaluate and broaden the results of the logical analysis. Thus, a crucial advantage of our approach is the ability to incorporate experimental data from heterogeneous sources, carrying qualitative, semi-quantitative or fully quantitative information. A further possibility would have been to take smaller steps on the modelling scale and add hybrid formalisms to the pipeline, e.g. discrete-continuous systems modelled as hybrid automata or Petri nets based on the boolean models that we derived in the first pipeline step [41, 45]. However, we found that in our context this does not provide any clear advantages, since the computational cost of parameter- and dynamic analysis that go beyond qualitative aspects is already close to that of an ODE model while still incorporating many abstractions of the underlying mechanisms [46, 47].

The ODE model, derived in the second step of the pipeline, allowed us to identify regions in the multidimensional kinetic parameter space inducing bistable behaviour. Using a Monte Carlo sampling procedure, we identified a sufficiently large amount of parameter sets inducing bistability in the wild type strain and monostable behaviour in particular knockout strains and strains with point mutations (see “Methods”). Note that sampling procedures are frequently used for identifying parameter regions inducing bistability [23, 48]. We post-hoc compared the identified parameter distributions with experimentally measured parameters in related systems. Thus, we could show that the network topology validated in the logical modelling step is capable of generating bistable dynamics within a biologically meaningful range of kinetic parameters, as shown in Fig. 3.

By conducting a bifurcation of key model parameters we exemplarily analysed the ranges where these parameters induce bistable regimes. This is of particular relevance since it was previously shown that during entry into the stationary phase of the growth cycle the *σ*^S^-controlled DGC enzyme YegE is induced, while the expression of the PDE enzyme YhjH (controlled by the flagellar master regulators FlhDC and FliA) is turned down. The remaining levels of the YhjH protein are diluted and degraded within the following cell divisions [8]. Our results suggest that by fine-tuning the levels of YegE and YhjH the cells maintain parametric configurations, which generate bistable behaviour. Stochastic simulations indicate that a further increase of the protein levels of YegE during the stationary phase might reduce the probability of the curli-off state and increase the probability of the curli-on state (see Fig. 4a, inlay plots). This is in line with experimental measurements of the distribution of curli cells in bacterial colonies. Serra *et al.* [11] showed a spatial variation of this distribution, where the relative amount of curli-on cells increases with an increasing vertical distance to the nutritional source at the bottom of the colony. In addition, Grantcharova *et al.* [10] observed a variation of this distribution within different stages of the stationary phase of the bacterial growth cycle. Furthermore, the bifurcation results suggested a narrow parameter region where the system is bistable. Changing these parameters in a way that eliminates the catalytic activity of the c-di-GMP-regulating enzymes YegE and YhjH, or decreases the binding affinity of c-di-GMP to YciR yielded monostable systems which could explain experimental results of corresponding single-gene knockout strains lacking *yegE* and *yhjH* or carrying a *yciR*^AAL^ point mutation (see Fig. 4).

Finally, we addressed a possible mechanism inducing spontaneous switching between different stable states. Stochastic simulations of the system indicated that depending on kinetic parameters, qualitatively different types of noise-induced switching dynamics may be observed, where most of the identified parameter sets indicated a higher probability of switching from the curli-off state to the curli-on state than vice versa (72 *%*). In line with these results, small chains of curli cells were observed within macrocolonies of *E. coli*, suggesting that once the cells switch to curli expression, they maintain this state and inherit it to successive cell generations [11]. We identified a significant subset of parameters (12 *%*) exhibiting a low switching probability in either direction (lower left corner in Fig. 5a). This suggests that certain parameter configurations might enable a long-term differentiation of the cells into curli producers and non-producers, retaining the status-quo until system parameters change again e.g. due to a variation of environmental conditions. A possible regulatory mechanism for controlling the switching behaviour might be given by the level of separation of steady states within distinct phenotypes. In support of this, we observed that the deviation of stable c-di-GMP levels between *curli-off* and *curli-on* phenotypes was much more pronounced in parameter sets that favour a differentiation (rare switchers) than those parameter sets that allow frequent switching between the two phenotypes. A greater separation of these levels may induce a larger (energetic) barrier and robustness against stochastic fluctuations, which could be a potential mechanism controlling the phenotypic plasticity of *E. coli*. A comparison of the steady state levels in Fig. 7 with experimentally measured numbers of system components, in particular c-di-GMP, in *curli-on* and *curli-off* cells may shed further light on this topic.

Note that in this study we only focused on the noise resulting from post-translational interactions. The implicit time-scale separation assumption can be justified by the longer time scales of fluctuations in protein copy numbers as compared to the longest simulations in this study (≤100 s). In our simulations, we observed that switching from curli-off to curli-on states was generally fast (≤100 s, see Fig. 5b) and may thus not be affected by slower noise processes, that are related to protein copy number variations. However, switching in the opposite direction (curli-on to curli-off) may occur at a slower time-scale (≥100s, see Fig. 5b) in some parameter sets and may thus be influenced by the noise resulting from transcriptional and translational events. Further kinetic data may enable a refinement of this model by explicitly including gene expression noise.

## Conclusion

In this study we have presented a framework for the analysis of multistable signalling networks with an application to the stationary phase-induced curli regulation system of *E. coli*. Using a hybrid sequential logical-continuous approach we first verified the potential of the curli regulation system for inducing bistability by incorporating genetic knockout experiments as discrete model constraints. Based on the validated network topology, we derived a reaction-rate model of the system and identified several parameter sets that are capable of inducing bistable dynamics. Notably, most identified parameter sets are located in a biologically meaningful region. In addition, we analysed the dependence of the probability of the stable curli-on and curli-off states on system parameters. We showed that certain parameter variations, e.g. the amount and activities of the DGCs and PDEs, give rise to different switching dynamics between curli-on and curli-off states (i.e. frequent switching or stable differentiation).

The present study introduces the first mathematical model of bistable regulation of curli fibers in *E. coli* based on post-translational interactions of multiple DGC and PDE proteins with the signalling molecule c-di-GMP as the central player. Our results deliver new potential targets for further experimental investigations, which may help to test the modelling hypotheses and to successively deepen the understanding of biofilm control in bacteria.

## Additional files

Additional file 1
**Supplementary information on logical modelling formalism including Figures S1 and Tables S1 and S2.**

Additional file 2
**Stepwise derivation of the reaction rates of the time-continuous model and description of the assumptions and of the validity criterion used for the derivation of the stochastic propensity functions.**

Additional file 3
**This.csv file contains the identified parameter sets used for the stochastic modelling of curli regulation in** ***E. coli*** **.**

Additional file 4
**Figure S2.** Violin plots showing the linearly scaled distribution of parameters in Fig. 3 of main manuscript along with the proposal distribution of the rejection sampling procedure (grey areas). The bounds of the proposal distribution, empirical means of each sampled parameter and the experimentally measured values of similar parameter values from the literature are equivalent to those in Fig. 3.

Additional file 5
**Figure S3.** Solutions of the Chemical Master Equation (Additional file 2: Eq. (S11)) based on Monte Carlo sampling [42]. Three different initial levels of c-di-GMP were used: *x*_1_=130,215 and 420 *molecules* (panel rows 1 to 3, respectively). The initial levels of the other two system variables were equally set to *x*_2_=0 (YciR in YdaM/MlrA inhibition state) and *x*_3_=768 (active YdaM). Three different simulation times *T* were used in order to identify the equilibration time of the system: *T*=20,100 and 200 seconds (panel columns 2 to 4). 10^3^ stochastic sample trajectories were generated for computing each solution. The final empirical sampling distributions in the figures were subject to a kernel smoothing.
