# Supplementary material for: Logical-continuous modelling of post-translationally regulated bistability of curli fiber expression in Escherichia coli
Source: BMC Syst Biol. 2015 Jul 23;9:39. doi: 10.1186/s12918-015-0183-x (PMC4511525; doi:10.1186/s12918-015-0183-x)
Supplement: Additional file 1 — Supplementary information on logical modelling formalism including Figures S1 and Tables S1 and S2. [file 12918_2015_183_MOESM1_ESM.pdf]

Details on logical model and logical data analysis

The logical modelling step is performed to test whether the putative model topology (Fig.1B, main manuscript) is capable of inducing bistability and fulfilling all observations (constraints) given by the experimental knock-out data (Fig.1A, main manuscript). To this end, the entire set of parametrizations (denoted $\mathcal{K}$, see further) of regulatory functions for the *components* (YdaM, c-di-GMP, etc...) is scanned and it is tested whether each of these parametrizations $K \in \mathcal{K}$ can fulfil all observations from the biological knock-out experiments. This model checking procedure then results in a set of valid parametrizations $\mathcal{K}^*$. Lastly, the set of valid parametrizations is further analysed, which allows for model simplifications and the derivation of parameter constraints for the subsequent time-continuous modelling step. In this section we provide the full formal description of the employed logical framework and a formal version of the logical model we built in the *Methods* section. We also list all formal properties, the full description of the results and lastly elaborate on the methods used for parameter set analysis.

Formalism and set-up

In the following we describe the logical modelling framework. While the notation is slightly modified to better suit our modelling aim, there is no practical difference to the standard framework of multi-valued logical models of René Thomas as described for example in [3].

State space

We denote V to be a set of named *components*. In the main text we stated that components v are either *on* or *off*, except for YciR, which has three activity levels. In the following, we will identify the *off* state with the value 0 and the *on* state with 1. For YciR, the values 2 and 1 correspond to the c-di-GMP degradation activity and the YdaM/MlrA inhibition activity, respectively.
More generally, each component can take integer values $n \in [0, \rho(v)]$, where $\rho : V \to \mathbb{N}^+$ assigns a maximal *activity level* to each component, see Figure S1(a). Since the range of values for each component is finite, we can describe the set of all possible configurations of the system, the logical *state space*, as $S = \prod_{v \in V}[0, \rho(v)]$. The set S therefore represents all qualitatively different configurations of a system (combinations of logical values for the distinct species, e.g. *on* and *off*). In the following, $s \in S$ refers to a particular state of the system and by $s_v = n$ we state that the component v of the state s has activity level n.

Space of regulatory functions

Edges. In the logical modelling context, each component v depends only on the values of its regulators $u \in V$. We denote by $E \subseteq V \times \mathbb{N}^+ \times V$, $n \leq \rho(u)$ for all $(u, n, v) \in E$ a set of *regulations* (regulatory edges), such that $G = (V, E, \rho)$ denotes a multi-valued regulatory network. We create a labelling function $l : E \to \{\text{Activating Only}, \text{Inhibiting Only}, \text{Free}\}$. The formal definition is provided later, but intuitively, Activating Only edges assign the activity value of the regulator u to the regulated node v, whereas Inhibiting Only edges assign the opposing activity level. Free edges have no requirements. The set of regulatory edges is depicted in Fig. S1(b).
Thresholds and regulatory contexts. We use the function $\theta : V \times V \to 2^{\mathbb{N}^+}$ that provides the *thresholds* of all edges between two vertices and which is defined as $\theta(u, v) = \{n \mid (u, n, v) \in E\}$ where $u, v \in V$. Note that $\theta(u, v) = \emptyset$ if there is no edge from u to v. Thresholds divide the *activity intervals* of a regulator, with respect to the effect that they exert on the regulated component (e.g. activation), defined as:

$$I_v^u = \{[j, k) \mid j, k \in \theta(u, v) \cup \{0, \rho(u) + 1\}, j < k, \neg\exists l \in \theta(u, v) : j < l < k\}. \quad (1)$$

Note that $\bigcup I_v^u = [0, \rho(u)]$, even in the case that there is no edge from u to v.
A *regulatory context* of a component $v \in V$—a set of all the possible combinations of activities of its regulators—is denoted and defined as $\Omega_v = \prod_{u \in V} I_v^u$.
Parameterizations. The dynamics of a component v based on the activities of its regulators denotes

activity levels	
component v	max. act. $\rho(v)$
YegE	1
YhjH	1
c-di-GMP	1
YciR	2
YdaM	1
MlrA	1

(a)

regulatory edges			
$u \in V$	$n \in [1, \rho(u)]$	$v \in V$	$l(u, n, v)$
YhjH	1	YhjH	Activating Only
YegE	1	YegE	Activating Only
YhjH	1	c-di-GMP	Inhibiting Only
YegE	1	c-di-GMP	Activating Only
YdaM	1	c-di-GMP	Activating Only
YciR	2	c-di-GMP	Inhibiting Only
c-di-GMP	1	YciR	Free
YciR	1	YdaM	Inhibiting Only
YciR	2	YdaM	Free
YciR	1	MlrA	Inhibiting Only
YciR	2	MlrA	Free
YdaM	1	MlrA	Activating Only

(b)

$\forall s \in \mathcal{S}, s_{\text{C-di-GMP}} = 1 : K_{\text{YciR}}(s) = 2$

$\forall s \in \mathcal{S}, s_{\text{C-di-GMP}} = 0 : K_{\text{YciR}}(s) = 1$

$\forall s, s' \in \mathcal{S}, s_{\text{YciR}} = 0, s' = s[\text{YciR}/2] : (K_{\text{MlrA}}(s) = K_{\text{MlrA}}(s')) \wedge (K_{\text{YdaM}}(s) = K_{\text{YdaM}}(s'))$

$\forall s, s' \in \mathcal{S}, s_{\text{YciR}} = 0, s' = s[\text{YciR}/1] : K_{\text{c-di-GMP}}(s) = K_{\text{c-di-GMP}}(s')$

(c)

Figure S1: **Formal description of the logical regulatory graph.** (a) The components v and their respective maximum activity levels $\rho(v)$. (b) Regulators u acting in their activity levels n on components v. The edge labels $l(u, n, v)$ indicate that regulatory edges are constrained to particular regulatory sets, i.e. sets of 'activating' or 'inhibiting' regulations. Additional constraints (c) are imposed on the regulation of and by YciR to achieve the required semantics. The term $s[v/n]$ where $s \in \mathcal{S}, v \in V, n \in \mathbb{N}$ denotes a state s where the value of the index v has been replaced by n, e.g. the first constraint states that if c-di-GMP is in state 1 ('*on*'), then YciR is updated from state 1 to state 2.

its *regulatory function.* The regulatory function of a component is defined as $K_v : \Omega_v \to [0, \rho(v)]$ for each component $v \in V$. A vector of regulatory functions for each component $K = (K_v)_{v \in V}$ is called a *parametrization.* Each parametrization uniquely defines the dynamics of a regulatory network, as further explained, and we use the term *model* for a pair (G, K) where K is a parametrization of the regulatory network G.

Note that there are usually many possible regulatory functions for a component $v \in V$, the set of which is denoted $\mathcal{K}_v$. The product of these $\mathcal{K} = (\mathcal{K}_v)_{v \in V}$ is then called the *parametrization space* and it naturally corresponds to a set of all possible logical models.

Update functions. Having a regulatory graph $G = (V, E, \rho)$ and a parametrization K we can fully describe its dynamical behaviour as a *transition system* over its state space S. This is a directed graph $(S, \to)$ where $\to \subset S \times S$ is the *transition relation.* Here, we are interested in asynchronous dynamics, which means that the transition relation is non-deterministic.

To obtain the relation $\to$, K is converted into an *update function* $F^K = (F_v^K)_{v \in V}$ where $F_v^K : S \to [0, \rho(v)]$ for all $v \in V$. Here we exploit the fact that for each $s \in S$ and for each $v \in V$ there exists a context $\omega \in \Omega_v$ such that $s \in \prod_{u \in V} \omega_u$. To simplify the notation we will further write $s \in \omega$ instead of $s \in \prod_{u \in V} \omega_u$. For

every $v \in V$, we obtain the function F_v^K from the parametrization by setting

$$F_v^K(s) = \begin{cases} s_v + 1, & \text{if } s_v < K_v(\omega), s \in \omega, \\ s_v, & \text{if } s_v = K_v(\omega), s \in \omega, \\ s_v - 1, & \text{if } s_v > K_v(\omega), s \in \omega. \end{cases} \quad (2)$$

Note that in our context, YegE and YhjH are treated as inputs and always keep the value they were initially assigned. This is achieved by putting positive feedback loops on each of these two *components*.

Edge constraints. The edge labels as described above are resolved on the parametrizations. Given an edge (u, n, v) such that $[n_-, n), [n, n_+) \in I_v^u$ for some $n_-, n_+ \in [0, \rho(u)]$. Then (u, n, v) is Activating *iff* there exists $\omega \in \Omega_v$ such that $\omega_u = [n_-, n)$ and $K_v(\omega) < K_v(\omega[u/[n, n_+)])$. Likewise (u, n, v) is Inhibiting *iff* there exists $\omega \in \Omega_v$ such that $\omega_u = [n_-, n)$ and $K_v(\omega) > K_v(\omega[u/[n, n_+)])$. An edge is Activating Only *iff* it is Activating and not Inhibiting. An edge is Inhibiting Only *iff* it is Inhibiting and not Activating. A Free edge has no requirements.

Additional constraints. Usually, an increase of the state value of a component represents an increase in the concentration of the respective species. Here this is not the case as our assumption is that the total amount of YciR is conserved. Our choice (which value represents the particular situation) is arbitrary and has no impact on the results. To model this situation, we explicitly set the parameters, i.e., formulate the rules, for regulation by c-di-GMP as shown in Figure S1c. There, we set YciR to level 2 if c-di-GMP is present and to level 1 if it is not. Also, we had to explicitly specify that YciR regulates c-di-GMP only at the level 2 and YdaM and MlrA only at the level 1 in line with the interpretation of the values, which is also formalized in Figure S1(c).

Transition system analysis

Having F^K, we now assign each parametrized regulatory graph a transition system via the function $T : \mathcal{K} \to \{(S, \to)\}$ where $T(K) = (S, \to)$ such that

$$\forall v \in V, \forall s \in S (s \to s[v/n] \iff (F_v^K(s) = n \wedge F_v^K(s) \neq s_v)), \quad (3)$$

with $x[i/k]$ denoting that in the vector x, the i-th value is substituted for k. Lastly, for the model analysis we use the LTL model checking [1] technique. We encode the experimental observations as a set of logical properties $\varphi = \{\varphi_1, \dots\}$ (exemplified below) and require that any valid model exhibits these properties.

In the transition system $(S, \to)$ we label *component* v of each state s with its activity level, i.e. $s_v = n$. Additionally, we add a label SS to all stable state (all states for which it holds that there is no $s' \in S$ such that $s \to s'$). For all experiments we use the formulas of the form $\mathrm{G}(\text{knockouts}) \wedge (\text{initial state}) \wedge \mathrm{F}(\text{output} \wedge SS)$ where:

- G(knockouts) specifies the components that are globally (G) set to 0,
- (initial state) specifies the initial conditions of the experiment,
- F(output $\wedge SS$) specifies that in the future (F) the system will reach the state with the required output and remain there.

The formulas translate the experimental observations from the genetic knock-out experiments (Figure 1A, main manuscript) into the context of the logical model. Each knock-out experiment translates into two logical properties that are listed in Table S1. The set of parametrizations satisfying all properties is given in Table S2.

For illustration we detail the formal description for one of the observations. For experiment #4 (*ydaM* is knocked out), the *hyperrepressed* phenotype was observed. This observation translates into the two properties:

- $G(YdaM = 0) \wedge (MlrA = 0 \wedge YegE = 1 \wedge YhjH = 1) \wedge F(MlrA = 0 \wedge SS)$ - there is a trace from a state where YhjH and YegE is present and MlrA absent to a stable state where MlrA is absent, while YdaM is permanently absent (knocked out).

- $\neg(G(YdaM = 0) \wedge (MlrA = 0 \wedge YhjH = 1 \wedge YegE = 1) \wedge F(MlrA = 1 \wedge SS))$ - it is not true that there is a trace from a state where YhjH and YegE is present and MlrA absent to a stable state where MlrA is present, while YdaM is permanently absent (knocked out).

In the second step we test for each model whether it satisfies the properties via the automata based LTL model checking [1]. We convert a transition system $(\mathcal{S}, \rightarrow)$ into a so-called Kripke Structure by setting all the states $s \in S$ as both initial and final. We then check if the structure satisfies each of the properties. Formally, denote $\rightarrow^K$ the transition relation obtained from a parameterization $K \in \mathcal{K}$. We create the refined model set $\mathcal{K}'$ where $K \in \mathcal{K}' \iff \forall \varphi_i \in \varphi : \neg((\mathcal{S}, \rightarrow^K) \models \neg\phi_i)$. The check is conducted on a product of the Kripke Structure and a Büchi automaton for each of the properties. For more details please refer to the relevant literature [1].

The validation procedure yields 10 parametrizations (see Table S2) that satisfy the set of observations from the knock-out experiments. These 10 parametrizations refer to the interpretations of the data as stated in the *Methods* section of the main manuscript in subsection *Formalisation of the experimental data*.
Note that the observations from the knock-out experiments can also be interpreted in a less conservative sense: i.e. interpreting the data such that no curli is expressed in *both* the basal- and hyperrepressed phenotype (the stable state MlrA *off* is reachable, while the *on* state is not) and that the hyperactivated phenotype corresponds to the scenario where all cells express curli (the stable state MlrA *on* is reachable, but not the *off* state). In the latter scenario, there is one parameterization that fulfils the constraints arising from this less conservative interpretation of the experimental observations (see Fig.A, main manuscript). Thus, irrespective of the elaborated interpretation of the experimental observations in Fig. 1A (main manuscript), there exists at least one logical model that is consistent with the data. This parameterization is depicted in the first row of Table S2.

Analysis of results

In the *Results* section of the main manuscript we discuss several observations drawn from the final parametrization set $\mathcal{K}^*$ shown in Table S2. Here, we present our reasoning in more detail.

First, consider the regulation of MlrA. As seen in Table S2, there are two possible regulatory functions for MlrA (compare rows 1–5 with rows 6–10). All combinations of these two with the 5 possible functions for c-di-GMP yield the 10 valid models. Since our focus is on stabilizing behaviour, we distinguish parametrizations according to the stable states that they generate. If the network is to stabilize in a state where YciR exhibits its inhibiting effect on YdaM then YdaM needs to be *off*, since there are no other regulators influencing its activity. For the same reason, YciR switching to PDE activity must result in a change of activity level for YdaM, namely YdaM must become active. Consequently, in a stable state we either observe that YciR acts as an inhibitor and YdaM is *off*, or YciR exhibits its PDE activity and YdaM is *on*. Since MlrA is only regulated by YdaM and YciR, we know that in the first scenario MlrA needs to be *off*, since its inhibitor is active and its activator is inactive, and in the second, opposing scenario MlrA is *on*. That is, in a stable state the values of YdaM and MlrA always coincide and we can use YdaM as a marker for the curli production.

Furthermore, note that only one function for MlrA is possible (the function stated in rows 1–5 of Table S2) if we consider the constraint set relating to the less conservative interpretation of the knock-out experiments (see previous section). This is due to the interpretation of the basal phenotype as a biological state with no curli production, which is also the case when YdaM and YciR are both knocked out. In particular, this means that the absence of an inhibitory effect of YciR is not sufficient to initiate curli production and therefore eliminates one of the two previously valid MlrA regulatory functions. Since the logical framework is too coarse-grained to distinguish between differences in data that are of rather quantitative nature (as might be the case when considering the basal and the hyperrepressed phenotype), the underlying assumptions and consequently the result of this analysis have to be carefully evaluated in the biological context.

As a further result, we identified YdaM as the most prominent regulator of c-di-GMP. Let us recall the assumption that the pool of c-di-GMP is shared between YegE, YciR, YdaM, and YhjH. We assume that there are no competitive reactions between the above listed four proteins on any single molecule of c-di-GMP, meaning that c-di-GMP is active in the system if and only if the production/degradation ratio stabilizes in a state with a sufficiently high concentration of c-di-GMP. From the final parametrization set, we can see that the conditions for c-di-GMP to be *on* are either that the activating effect exhibited by YdaM is already sufficient on its own (see parameterization sets corresponding to row 4 & 9 in Table S2), or only one other activating influence (YegE being *on*, YhjH being *off* or YciR being at its MlrA/YdaM inhibitor activity) is needed (see the remaining parametrizations/rows of Table S2). Conversely, if the activating influence of YdaM is absent, this cannot be compensated (the parameter sets in rows 2 & 7 in Table S2), or only if one activating influence is combined with the absence of at least one inhibiting influence on c-di-GMP (the remaining rows in Table S2).

Continuing on the idea of functional independence we can qualitatively compare the pair-wise effects of competitive regulators of c-di-GMP. Two observations appear:

1. YdaM is stronger than YciR: in all parametrizations we observe that when YdaM is *on*, YciR is at its PDE activity, and the inputs (YhjH and YegE) are *off* then c-di-GMP is *on*.
2. YhjH is stronger than YegE: in all parametrizations we observe that when only YhjH and YegE are *on* then c-di-GMP is *off*.

Such observations can now be exploited to derive constraints for the parameter sampling for the ODE model.

In the following, we will only focus on the observation 1). As a first step we translate the logical scenario for our observation concerning c-di-GMP regulation into the continuous setting. Since in the statement 1) YegE and YhjH are in the off configuration (e.g. knocked out), we can drop the first two terms in the ODE describing c-di-GMP behaviour, meaning the equation

$$\frac{d}{dt}x_1 = \frac{V_{\max 1}}{1 + x_1/K_{\mathrm{i}}^{\mathrm{YegE}}} - \frac{V_{\max 2}x_1}{x_1 + K_{\mathrm{m}}^{\mathrm{YhjH}}} - (YciRtot - x_2)\frac{k_{\mathrm{YciRact}}x_1}{x_1 + K_{\mathrm{m}}^{\mathrm{YciR}}} + \frac{k_{\mathrm{YdaMact}}x_3^n}{(K_{\mathrm{d_{polymer}}}^{\mathrm{ydaM}})^n + x_3^n},$$

simplifies to:

$$\frac{d}{dt}x_1 = -(YciRtot - x_2)\frac{k_{\mathrm{YciRact}}x_1}{x_1 + K_{\mathrm{m}}^{\mathrm{YciR}}} + \frac{k_{\mathrm{YdaMact}}x_3^n}{(K_{\mathrm{d_{polymer}}}^{\mathrm{ydaM}})^n + x_3^n}.$$

Much of the difficulty in relating a logical to an ODE model comes from the necessity to put *real-valued* and *discretized* values into relation, implying that we need to decide which ranges of *real* values correspond to the logical states of YdaM and c-di-GMP being *on* and YciR being in its PDE configuration. A variety of discretization methods are available, but the results often need to be carefully evaluated w.r.t. the application, see e.g. [2]. However, for our purposes we do not need to know the exact discretization thresholds, we just need to make sure that we evaluate the ODE at values for x_1, x_2 and x_3 that represent the logical values of c-di-GMP being *on*, YciR being in its PDE configuration (interpreted as YciR not acting as YdaM inhibitor) and YdaM being *on*. We achieve this by considering the limits $x_1 \to \infty, x_2 \to 0$, and $x_3 \to \infty$, which ensures that we crossed the respective discretization thresholds regardless of their actual value. Note that these limits can be interpreted as YciR being fully committed to PDE activity and YdaM and c-di-GMP fully saturating the corresponding rate functions. This results in the terms $x_1/(x_1 + K_{\mathrm{m}}^{\mathrm{YciR}})$ and $x_3^n/((K_{\mathrm{d_{polymer}}}^{\mathrm{ydaM}})^n + x_3^n)$ tending to the limit value 1. We obtain:

$$\begin{aligned}
\lim_{x_1\to\infty} \frac{k_{\mathrm{YciRact}}x_1}{x_1 + K_{\mathrm{m}}^{\mathrm{YciR}}} &= k_{\mathrm{YciRact}}, \\
\lim_{x_2\to 0} (YciRtot - x_2) &= YciRtot, \\
\lim_{x_3\to\infty} \frac{k_{\mathrm{YdaMact}}x_3^n}{(K_{\mathrm{d_{polymer}}}^{\mathrm{ydaM}})^n + x_3^n} &= k_{\mathrm{YdaMact}},
\end{aligned}$$

which we apply to our simplified ODE, obtaining the limit form:

$$\frac{d}{dt}x_1 = -\,YciRtot \cdot k_{\text{YciRact}} + k_{\text{YdaMact}}.$$

The target parameter for c-di-GMP in the corresponding logical states indicates that c-di-GMP remains active in this scenario. We now make the assumption that we can translate this into a constraint saying that the value of x_1 should not decrease (represented by $\frac{d}{dt}x_1 \geq 0$) whenever the system is in a biological state corresponding to the state of our logical scenario. In particular, this holds for our limit considerations. In general, this assumption will not always be true, since it is feasible that for large values inhibiting effects come into play that are again counteracted when approaching the discretization threshold. However, the logical model is set up in such a way that all qualitative regulation effects that could lead to such a behaviour are captured and represented by the different activity levels, so that we exclude the possibility of the observations not holding asymptotically. This is also in agreement with the biological interpretation of those limits mentioned above. Inserting the condition for $\frac{d}{dt}x_1$ into the ODE then yields the inequality

$$0 \leq -\,YciRtot \cdot k_{\text{YciRact}} + k_{\text{YdaMact}},$$

giving us the constraint:

$$YciRtot \cdot k_{\text{YciRact}} \leq k_{\text{YdaMact}}.$$

To further ensure that our assumptions are proper, we relax this constraint based on the following observation. $YciRtot$ represents the total number of YciR molecules in the system. We know that YciR is present, therefore $YciRtot \geq 1$ and from there:

$$k_{\text{YciRact}} \leq YciRtot \cdot k_{\text{YciRact}} \leq k_{\text{YdaMact}}.$$

Since the value for $YciRtot$ is expected to be much larger than 1, we can utilize the much weaker constraint

$$k_{\text{YciRact}} \leq k_{\text{YdaMact}}.$$

with high confidence.

Similar arguments can be made to derive a constraint from the observation 2) listed above. However, while the formal steps can still be executed, the biological interpretation becomes more difficult, and the logical observation is more local as in the previous case where the strong impact of YdaM was supported by a global analysis. For these reasons we decided not to include the corresponding constraint in the parameter sampling procedure stated in the *Methods* section of the main manuscript.

References

[1] C. Baier and J.-P. Katoen. *Principles of Model Checking*. The MIT Press, 2008.

[2] E. S. Dimitrova, M. P. V. Licona, J. McGee, and R. Laubenbacher. Discretization of time series data. *Journal of Computational Biology*, 17(6):853–868, 2010.

[3] R. Thomas. Regulatory networks seen as asynchronous automata: a logical description. *Journal of theoretical biology*, 153(1):1–23, 1991.

knock-out #	LTL formula
**1**	$(\text{YegE} = 1 \wedge \text{YhjH} = 1 \wedge \text{MlrA} = 0) \wedge \text{F}(\text{MlrA} = 1 \wedge SS)$
**1**	$(\text{YegE} = 1 \wedge \text{YhjH} = 1 \wedge \text{MlrA} = 0) \wedge \text{F}(\text{MlrA} = 0 \wedge SS)$
**2**	$\text{G}(YciR = 0) \wedge (\text{MlrA} = 0 \wedge \text{YegE} = 1 \wedge \text{YhjH} = 1) \wedge \text{F}(\text{MlrA} = 1 \wedge SS)$
2	$\neg(\text{G}(YciR = 0) \wedge (\text{MlrA} = 0 \wedge \text{YegE} = 1 \wedge \text{YhjH} = 1) \wedge \text{F}(\text{MlrA} = 0 \wedge SS))$
**3**	$\text{G}(\text{YhjH} = 0) \wedge (\text{MlrA} = 0 \wedge \text{YegE} = 1) \wedge \text{F}(\text{MlrA} = 1 \wedge SS)$
3	$\neg(\text{G}(\text{YhjH} = 0) \wedge (\text{MlrA} = 0 \wedge \text{YegE} = 1) \wedge \text{F}(\text{MlrA} = 0 \wedge SS))$
**4**	$\neg(\text{G}(\text{YdaM} = 0) \wedge (\text{MlrA} = 0 \wedge \text{YegE} = 1 \wedge \text{YhjH} = 1) \wedge \text{F}(\text{MlrA} = 1 \wedge SS))$
**4**	$\text{G}(\text{YdaM} = 0) \wedge (\text{MlrA} = 0 \wedge \text{YegE} = 1 \wedge \text{YhjH} = 1) \wedge \text{F}(\text{MlrA} = 0 \wedge SS)$
5	$\neg(\text{G}(\text{YegE} = 0) \wedge (\text{YhjH} = 1 \wedge \text{MlrA} = 0) \wedge \text{F}(\text{MlrA} = 1 \wedge SS))$
5	$\text{G}(\text{YegE} = 0) \wedge (\text{YhjH} = 1 \wedge \text{MlrA} = 0) \wedge \text{F}(\text{MlrA} = 0 \wedge SS)$
**6**	$\text{G}(\text{YciR} = 0 \wedge \text{YhjH} = 0) \wedge (\text{YegE} = 1 \wedge \text{MlrA} = 0) \wedge \text{F}(\text{MlrA} = 1 \wedge SS)$
6	$\neg(\text{G}(\text{YciR} = 0 \wedge \text{YhjH} = 0) \wedge (\text{YegE} = 1 \wedge \text{MlrA} = 0) \wedge \text{F}(\text{MlrA} = 0 \wedge SS))$
7	$\neg(\text{G}(\text{YciR} = 0 \wedge \text{YdaM} = 0) \wedge (\text{YegE} = 1 \wedge \text{YhjH} = 1 \wedge \text{MlrA} = 0) \wedge \text{F}(\text{MlrA} = 1 \wedge SS))$
7	$\text{G}(\text{YciR} = 0 \wedge \text{YdaM} = 0) \wedge (\text{YegE} = 1 \wedge \text{YhjH} = 1 \wedge \text{MlrA} = 0) \wedge \text{F}(\text{MlrA} = 0 \wedge SS)$
**8**	$\text{G}(\text{YegE} = 0 \wedge \text{YciR} = 0) \wedge (\text{YhjH} = 1 \wedge \text{MlrA} = 0) \wedge \text{F}(\text{MlrA} = 1 \wedge SS)$
8	$\neg(\text{G}(\text{YegE} = 0 \wedge \text{YciR} = 0) \wedge (\text{YhjH} = 1 \wedge \text{MlrA} = 0) \wedge \text{F}(\text{MlrA} = 0 \wedge SS))$
**9**	$\text{G}(\text{YegE} = 0 \wedge \text{YhjH} = 0) \wedge \text{MlrA} = 0 \wedge \text{F}(\text{MlrA} = 1 \wedge SS)$
**9**	$\text{G}(\text{YegE} = 0 \wedge \text{YhjH} = 0) \wedge \text{MlrA} = 0 \wedge \text{F}(\text{MlrA} = 0 \wedge SS)$
10	$\neg(\text{G}(\text{YdaM} = 0 \wedge \text{YhjH} = 0) \wedge (\text{YegE} = 1 \wedge \text{MlrA} = 0) \wedge \text{F}(\text{MlrA} = 1 \wedge SS))$
10	$\text{G}(\text{YdaM} = 0 \wedge \text{YhjH} = 0) \wedge (\text{YegE} = 1 \wedge \text{MlrA} = 0) \wedge \text{F}(\text{MlrA} = 0 \wedge SS)$
**11**	$\neg(\text{G}(\text{YdaM} = 0 \wedge \text{YegE} = 0) \wedge (\text{YhjH} = 1 \wedge \text{MlrA} = 0) \wedge \text{F}(\text{MlrA} = 1 \wedge SS))$
**11**	$\text{G}(\text{YdaM} = 0 \wedge \text{YegE} = 0) \wedge (\text{YhjH} = 1 \wedge \text{MlrA} = 0) \wedge \text{F}(\text{MlrA} = 0 \wedge SS)$
12	$\neg(\text{G}(\text{YciR} = 0 \wedge \text{YdaM} = 0 \wedge \text{YhjH} = 0) \wedge (\text{YegE} = 1 \wedge \text{MlrA} = 0) \wedge \text{F}(\text{MlrA} = 1 \wedge SS))$
12	$\text{G}(\text{YciR} = 0 \wedge \text{YdaM} = 0 \wedge \text{YhjH} = 0) \wedge (\text{YegE} = 1 \wedge \text{MlrA} = 0) \wedge \text{F}(\text{MlrA} = 0 \wedge SS)$
**13**	$\text{G}(\text{YciR} = 0 \wedge \text{YegE} = 0 \wedge \text{YhjH}) \wedge \text{MlrA} = 0 \wedge \text{F}(\text{MlrA} = 1 \wedge SS)$
13	$\neg(\text{G}(\text{YciR} = 0 \wedge \text{YegE} = 0 \wedge \text{YhjH}) \wedge \text{MlrA} = 0 \wedge \text{F}(\text{MlrA} = 0 \wedge SS))$
14	$\neg(\text{G}(\text{YciR} = 0 \wedge \text{YdaM} = 0 \wedge \text{YegE} = 0) \wedge (\text{YhjH} = 1 \wedge \text{MlrA} = 0) \wedge \text{F}(\text{MlrA} = 1 \wedge SS))$
14	$\text{G}(\text{YciR} = 0 \wedge \text{YdaM} = 0 \wedge \text{YegE} = 0) \wedge (\text{YhjH} = 1 \wedge \text{MlrA} = 0) \wedge \text{F}(\text{MlrA} = 0 \wedge SS)$
**15**	$\neg(\text{G}(\text{YdaM} = 0 \wedge \text{YegE} = 0 \wedge \text{YhjH} = 0) \wedge \text{MlrA} = 0 \wedge \text{F}(\text{MlrA} = 1 \wedge SS))$
**15**	$\text{G}(\text{YdaM} = 0 \wedge \text{YegE} = 0 \wedge \text{YhjH} = 0) \wedge \text{MlrA} = 0 \wedge \text{F}(\text{MlrA} = 0 \wedge SS)$
16	$\neg(\text{G}(\text{YdaM} = 0 \wedge \text{YciR} = 0 \wedge \text{YegE} = 0 \wedge \text{YhjH} = 0) \wedge \text{MlrA} = 0 \wedge \text{F}(\text{MlrA} = 1 \wedge SS))$
16	$\text{G}(\text{YdaM} = 0 \wedge \text{YciR} = 0 \wedge \text{YegE} = 0 \wedge \text{YhjH} = 0) \wedge \text{MlrA} = 0 \wedge \text{F}(\text{MlrA} = 0 \wedge SS)$

Table S1: **The list of all LTL formulas used in model checking.** Each observations from the genetic knock-out experiments translates into two LTL properties. Note that the numbering of the experimental conditions corresponds to their appearance (from top to bottom) in Figure 1A (main manuscript). I.e. the first two lines correspond to the wild type, line 3 and 4 to the YciR knock-out, etc.... The 15 properties, which relate to the observations from the genetic knock-out data and the interpretation of the data as stated in the *Methods* section of the main manuscript in section *Formalisation of the experimental data* are highlighted by bold fonts (first column). The remaining properties are only valid if the experimental data is interpreted in a less conservative sense (see last paragraph before *Analysis of results* within this document.)

$K_{\text{YdaM}}(s)$	$K_{\text{YciR}}(s)$	$K_{\text{MlrA}}(s)$	
**$s_{\text{YciR}} = 0 \vee s_{\text{YciR}} = 2$**	**$(s_{\text{c-di-GMP}} = 1) + 1$**	**$(s_{\text{YciR}} = 0 \vee s_{\text{YciR}} = 2) \wedge s_{\text{Ydam}} = 1$**	
$s_{\text{YciR}} = 0 \vee s_{\text{YciR}} = 2$	$(s_{\text{c-di-GMP}} = 1) + 1$	$(s_{\text{YciR}} = 0 \vee s_{\text{YciR}} = 2) \wedge s_{\text{Ydam}} = 1$	
$s_{\text{YciR}} = 0 \vee s_{\text{YciR}} = 2$	$(s_{\text{c-di-GMP}} = 1) + 1$	$(s_{\text{YciR}} = 0 \vee s_{\text{YciR}} = 2) \wedge s_{\text{Ydam}} = 1$	
$s_{\text{YciR}} = 0 \vee s_{\text{YciR}} = 2$	$(s_{\text{c-di-GMP}} = 1) + 1$	$(s_{\text{YciR}} = 0 \vee s_{\text{YciR}} = 2) \wedge s_{\text{Ydam}} = 1$	
$s_{\text{YciR}} = 0 \vee s_{\text{YciR}} = 2$	$(s_{\text{c-di-GMP}} = 1) + 1$	$(s_{\text{YciR}} = 0 \vee s_{\text{YciR}} = 2) \wedge s_{\text{Ydam}} = 1$	···
$s_{\text{YciR}} = 0 \vee s_{\text{YciR}} = 2$	$(s_{\text{c-di-GMP}} = 1) + 1$	$(s_{\text{YciR}} = 0 \vee s_{\text{YciR}} = 2) \vee s_{\text{Ydam}} = 1$	
$s_{\text{YciR}} = 0 \vee s_{\text{YciR}} = 2$	$(s_{\text{c-di-GMP}} = 1) + 1$	$(s_{\text{YciR}} = 0 \vee s_{\text{YciR}} = 2) \vee s_{\text{Ydam}} = 1$	
$s_{\text{YciR}} = 0 \vee s_{\text{YciR}} = 2$	$(s_{\text{c-di-GMP}} = 1) + 1$	$(s_{\text{YciR}} = 0 \vee s_{\text{YciR}} = 2) \vee s_{\text{Ydam}} = 1$	
$s_{\text{YciR}} = 0 \vee s_{\text{YciR}} = 2$	$(s_{\text{c-di-GMP}} = 1) + 1$	$(s_{\text{YciR}} = 0 \vee s_{\text{YciR}} = 2) \vee s_{\text{Ydam}} = 1$	
$s_{\text{YciR}} = 0 \vee s_{\text{YciR}} = 2$	$(s_{\text{c-di-GMP}} = 1) + 1$	$(s_{\text{YciR}} = 0 \vee s_{\text{YciR}} = 2) \vee s_{\text{Ydam}} = 1$	

$K_{\text{c-di-GMP}}(s)$
**$((s_{\text{YciR}} < 2 \vee s_{\text{YhjH}} = 0 \vee s_{\text{YegE}} = 1) \wedge s_{\text{YdaM}} = 1) \vee (s_{\text{YhjH}} = 0 \wedge s_{\text{YegE}} = 1)$**
$(s_{\text{YciR}} < 2 \vee s_{\text{YhjH}} = 0 \vee s_{\text{YegE}} = 1) \wedge (s_{\text{YdaM}} = 1)$
$((s_{\text{YciR}} < 2 \vee s_{\text{YhjH}} = 0 \vee s_{\text{YegE}} = 1) \wedge s_{\text{YdaM}} = 1) \vee (s_{\text{YciR}} < 2 \wedge s_{\text{YhjH}} = 0 \wedge s_{\text{YegE}} = 1)$
$(s_{\text{YdaM}} = 1) \vee (s_{\text{YciR}} < 2 \wedge s_{\text{YhjH}} = 0 \wedge s_{\text{YegE}} = 1)$
$((s_{\text{YhjH}} = 0 \vee s_{\text{YegE}} = 1) \wedge s_{\text{YdaM}} = 1) \vee (s_{\text{YciR}} < 2 \wedge s_{\text{YhjH}} = 0 \wedge s_{\text{YegE}} = 1)$
$((s_{\text{YciR}} < 2 \vee s_{\text{YhjH}} = 0 \vee s_{\text{YegE}} = 1) \wedge s_{\text{YdaM}} = 1) \vee (s_{\text{YhjH}} = 0 \wedge s_{\text{YegE}} = 1)$
$(s_{\text{YciR}} < 2 \vee s_{\text{YhjH}} = 0 \vee s_{\text{YegE}} = 1) \wedge (s_{\text{YdaM}} = 1)$
$((s_{\text{YciR}} < 2 \vee s_{\text{YhjH}} = 0 \vee s_{\text{YegE}} = 1) \wedge s_{\text{YdaM}} = 1) \vee (s_{\text{YciR}} < 2 \wedge s_{\text{YhjH}} = 0 \wedge s_{\text{YegE}} = 1)$
$(s_{\text{YdaM}} = 1) \vee (s_{\text{YciR}} < 2 \wedge s_{\text{YhjH}} = 0 \wedge s_{\text{YegE}} = 1)$
$((s_{\text{YhjH}} = 0 \vee s_{\text{YegE}} = 1) \wedge s_{\text{YdaM}} = 1) \vee (s_{\text{YciR}} < 2 \wedge s_{\text{YhjH}} = 0 \wedge s_{\text{YegE}} = 1)$

Table S2: **The set of parametrizations $\mathcal{K}^*$ whose respective transition system $T(K)$ where $K \in \mathcal{K}^*$ satisfies all logical properties.** Each parametrization shown in the table had to fulfill the 15 logical properties stated in Table S1 (bold indices), which relate to the observations from the genetic knock-out data and the interpretation of the data as stated in the *Methods* section of the main manuscript in section *Formalisation of the experimental data*. Note that the observations from the knock-out experiments can also be interpreted in a less conservative sense: i.e. requiring that no curli is expressed in both the basal- and hyperrepressed phenotype (the stable state MlrA *off* is reachable, while the *on* state is not) and requiring that the hyperactivated phenotype corresponds to the scenario where all cells express curli (the stable state MlrA *on* is reachable, but not the *off* state). In the latter scenario, there is one parameterization that fulfils the constraints arising from this less conservative interpretation of the experimental observations (see Fig.A, main manuscript). This parameterization is highlighted in blue (first row of this table). To simplify the notation we describe the functions via expressions of Boolean algebra, meaning that an expression evaluates to 1 if it is true and to 0 otherwise. The components YegE and YhjH are input components and therefore not listed (For an input component c it holds that $K_c(s) = s_c$ for all states $s \in S$).
