# Supplementary material for: Logical-continuous modelling of post-translationally regulated bistability of curli fiber expression in Escherichia coli
Source: BMC Syst Biol. 2015 Jul 23;9:39. doi: 10.1186/s12918-015-0183-x (PMC4511525; doi:10.1186/s12918-015-0183-x)
Supplement: Additional file 2 — Stepwise derivation of the reaction rates of the time-continuous model and description of the assumptions and of the validity criterion used for the derivation of the stochastic propensity functions. [file 12918_2015_183_MOESM2_ESM.pdf]

Derivation of reaction rates for the continuous model

April 24, 2015

S1 Derivation of deterministic rates

In this section, we outline the derivation of deterministic rate laws for the continuous model depicted in Fig. 1d (main manuscript). In the following, molecular concentrations will be denoted by small italic letters, e.g. x_1 and chemical reaction symbols will be denoted by normal capital fonts e.g. $\mathrm{X_1}$.

S1.1 Catalytic reactions of c-di-GMP synthesis and degradation (R_1, R_2, R_3 and R_4)

S1.1.1 Degradation reactions R_2 and R_3

The degradation of c-di-GMP by a PDE-type enzyme can be generically described by the following reaction system:

$$\mathrm{E} + \mathrm{X_1} \underset{k_{-1}^{\mathrm{PDE}}}{\overset{k_1^{\mathrm{PDE}}}{\rightleftharpoons}} \mathrm{E{:}X_1} \xrightarrow{k_{\mathrm{cat}}^{\mathrm{PDE}}} \mathrm{P} + \mathrm{E}, \tag{S1}$$

where E denotes a molecule of the enzyme phosphodiesterase (PDE), $\mathrm{X_1}$ denotes a molecule of c-di-GMP and P is the product of this reaction, pGpG. Here, we made a quasi steady-state assumption for the formation of the $\mathrm{E{:}X_1}$-complex. This makes the product formation the rate limiting step, yielding the maximal catalytic velocity $V_{\max} = (e + ex_1) \cdot k_{\mathrm{cat}}^{\mathrm{PDE}}$ and the Michaelis-Menten constant $K_{\mathrm{m}} = (k_{-1}^{\mathrm{PDE}} + k_{\mathrm{cat}}^{\mathrm{PDE}})/k_1^{\mathrm{PDE}}$ [1].

The two important PDEs controlling the curli regulation system are YhjH and YciR. Similarly to the DGC enzyme YegE, the amount of active YhjH proteins in the system is assumed to be constant. Therefore, the Michaelis-Menten reaction rate of YhjH results in

$$V_2 = \frac{V_{\max 2} x_1}{x_1 + K_{\mathrm{m}}^{\mathrm{YhjH}}}, \tag{S2}$$

where x_1 denotes the concentration of c-di-GMP and $K_{\mathrm{m}}^{\mathrm{YhjH}}$ is the Michaelis-Menten constant of YhjH and $V_{\max 2}$ is the corresponding maximal catalytic velocity.

The degradation of c-di-GMP by YciR has a similar form as eq. (S2). However, we explicitly modelled the amount of YciR in its catalytic form $\tilde{x}_2$ (YciR I in Fig. 1B of the main manuscript). This pool of YciR sums up with the pool of non-catalytic YciR (x_2) to $\tilde{x}_2 + x_2 = \mathit{YciRtot}$. As a result, the Michaelis-Menten reaction rate of YciR, V_3, is modelled by

$$V_3 = (\mathit{YciRtot} - x_2)\frac{k_{\text{YciRact}} x_1}{x_1 + K_{\text{m}}^{\text{YciR}}}, \tag{S3}$$

where ($\mathit{YciRtot}$–x_2) denotes the amount of catalytically active YciR molecules. By assuming the relation $k_1^{\text{PDE}} \ll k_{-1}^{\text{PDE}}$ this quantity can be set equal to the total amount of catalytically active enzyme (bound and unbound by c-di-GMP). Furthermore, k_{YciRact} models the rate-limiting product formation step and thus corresponds to the rate parameter $k_{\text{cat}}^{\text{PDE}}$ in the generic reaction scheme (S1).

S1.1.2 Synthesis reactions R_1 and R_4

The synthesis reaction of c-di-GMP is based on the transformation of two GTP substrate molecules into one molecule of c-di-GMP and 2 PPi molecules. The mechanistic details of this reaction were elucidated for several DGC-type enzymes [2, 3]. Within the curli regulation system the two DGC enzymes YegE and YdaM are assumed to play an important role. In contrast to YdaM, the reaction of YegE is subject to non-competitive inhibition (see e.g. [1]), which is induced by binding of the product molecule c-di-GMP to the I-site of the enzyme, inducing allosteric product (feedback) inhibition. Ultimately, this reaction generates c-di-GMP (X_1). Due to an excess availability of the substrate GTP [4] we assumed that all catalytic sites of the enzymes are occupied with substrate molecules GTP (i.e. $E_{\text{tot}} \approx E{:}S{:}S + E{:}S{:}S{:}X_1$), which reduces the reaction scheme to

$$\begin{array}{ccc} \mathrm{E{:}S{:}S} + \mathrm{X_1} & \xrightarrow{k_{\text{cat}}^{\text{DGC}}} & \mathrm{E} + 2\,\mathrm{X_1} \\ k_{\text{off}} \Big\uparrow\Big\downarrow k_{\text{on}} & & \\ \mathrm{E{:}S{:}S{:}X_1} & & \end{array} \tag{S4}$$

where for brevity of notation the formation of the PPi molecules as a byproduct of c-di-GMP synthesis is omitted. Since we assumed an excess availability of substrate, the synthesis reaction operates at maximal catalytic velocity $V_{\max 1}$, resulting in the kinetic rate V_1 given in the Table 1 of the main manuscript:

$$V_1 = \frac{V_{\max 1}}{1 + x_1/K_i}, \tag{S5}$$

where $K_i = k_{\mathrm{off}}/k_{\mathrm{on}}$ is the dissociation constant of c-di-GMP binding to the I-site of the enzyme. Further, we assumed that the product formation step is rate limiting in equation (S4). In contrast to YegE, the catalytic mechanism of YdaM is *not* subject to allosteric feedback inhibition [5]. However, YdaM molecules have been observed in a tetrameric form. For this reaction we assume complete homotropic cooperativity (see e.g. [1] for its definition), which necessitates to include a Hill parameter n. Again, we assume an excess availability of the substrate GTP, yielding the final rate function of YdaM shown in the Table 1 in the main manuscript:

$$V_4 = \frac{k_{\mathrm{YdaMact}} x_3^n}{\left(K_{\mathrm{d_{polymer}}}^{\mathrm{YdaM}}\right)^n + x_3^n}. \tag{S6}$$

where $K_{\mathrm{d_{polymer}}}^{\mathrm{YdaM}}$ denotes the microscopic dissociation constant and the variable x_3 denotes the concentration of YdaM.

S1.2 Regulatory reactions of protein activities

S1.2.1 Transition between catalytically active and inactive YciR (R_5, R_6)

It was previously shown that YciR changes its activity depending on the amount of c-di-GMP in the system [5]. Thus, a sufficiently high amount of c-di-GMP suppresses the inhibitory activity of YciR on YdaM and induces the catalytic activity of YciR leading to the degradation of c-di-GMP. Based on the observation that these two activities are mutually exclusive, we introduced two different states of this enzyme into our model: non-catalytically active YciR, with its pool denoted by the model variable x_2 (i.e. YciR II in Fig. 1 of the main manuscript) and YciR I, whose amount is given by $\tilde{x}_2$ =YciRtotal - x_2. The transformation of the YciR activity from YciR II to YciR I is most likely based on a specific interaction of YciR with c-di-GMP, which activates its catalytic activity as soon as there is a sufficiently high amount of c-di-GMP in the cell [5]. This reaction could e.g. be due to a c-di-GMP-induced conformation change. In order to model this interaction, we assumed a formation of an intermediate complex between YciR II and c-di-GMP, $\mathrm{x_1{:}x_2}$, which denotes a c-di-GMP bound YciR II molecule. The product of this reaction is $\tilde{\mathrm{x}}_2$ (YciR I). This gives rise to the following system of reactions:

$$R_5: \quad \mathrm{X_1 + X_2} \underset{k_{-1}^{\mathrm{R5}}}{\overset{k_1^{\mathrm{R5}}}{\rightleftharpoons}} \mathrm{X_1{:}X_2} \xrightarrow{k_{\mathrm{cat}}^{\mathrm{R5}}} \mathrm{X_1} + \tilde{\mathrm{X}}_2. \tag{S7}$$

By assuming that the formation of the enzyme-substrate complex occurs on a faster time scale than the product formation step and that $k_1^{\mathrm{R5}} \ll k_{-1}^{\mathrm{R5}}$ (i.e. $x_2 \gg x_1 : x_2$) and due to the conservation condition $YciRtot = x_2 + x_1{:}x_2 +$

$\tilde{x}_2$ we obtained a lumped reaction rate

$$V_5 = k_{\mathrm{YciRde}} x_2 \frac{x_1}{x_1 + K_{\mathrm{d}}^{\mathrm{YciR}}}, \tag{S8}$$

where $k_{\mathrm{YciRde}} = k_{\mathrm{cat}}^{\mathrm{R5}}$ and $(k_{-1}^{\mathrm{R5}} + k_{\mathrm{cat}}^{\mathrm{R5}})/k_1^{\mathrm{R5}} \approx k_{-1}^{\mathrm{R5}}/k_1^{\mathrm{R5}} = K_{\mathrm{d}}^{\mathrm{YciR}}$.

The reverse direction (YciR I $\rightarrow$ YciR II) was assumed to be given by a first order reaction:

$$R_6: \quad \tilde{\mathrm{X}}_2 \xrightarrow{c_6} \mathrm{X}_2.$$

This results in the reaction reaction rate

$$V_6 = c_6(YciRtot - x_2). \tag{S9}$$

S1.2.2 Inhibition reaction of YdaM by YciR II R_7 and reverse reaction R_8

We modelled the reactivation reaction of YdaM as a YciR-independent transformation of inactive YdaM to active YdaM

$$R_8: \quad \tilde{\mathrm{X}}_3 \xrightarrow{c_8} \mathrm{X}_3.$$

This first order reaction results in the following reaction rate

$$V_8 = c_8(YdaMtot - x_3).$$

The Inhibition of YdaM was suggested to be induced by binding to YciR II. In order to model this reaction, we assumed a multistep process

$$R_7: \quad \mathrm{X}_2 + \mathrm{X}_3 \underset{k_{-1}^{\mathrm{R7}}}{\overset{k_1^{\mathrm{R7}}}{\rightleftharpoons}} \mathrm{X}_2{:}\mathrm{X}_3 \xrightarrow{k_{\mathrm{cat}}^{\mathrm{R7}}} \mathrm{X}_2 + \tilde{\mathrm{X}}_3$$

where the formation of YciRII-YdaM complex $\mathrm{X}_2{:}\mathrm{X}_3$ is assumed to be fast and the inactivation step of YdaM is the rate-limiting step. Assuming that $\frac{d}{dt} x_2{:}x_3 \approx 0$ and $k_1^{\mathrm{R7}} \ll k_{-1}^{\mathrm{R7}}$ and $YdaMtot = x_3 + x_2{:}x_3 + \tilde{x}_3$ we obtain

$$x_2{:}x_3 \approx \frac{k_1^{\mathrm{R7}} x_2 x_3}{k_1^{\mathrm{R7}} x_2 + k_{-1}^{\mathrm{R7}} + k_{\mathrm{cat}}^{\mathrm{R7}}}.$$

This allows us to approximate the dynamics of this reaction by the following rate function

$$V_7 = k_{\mathrm{YdaMde}} x_3 \frac{x_2}{x_2 + K_{\mathrm{d}}^{\mathrm{YdaM}}} \tag{S10}$$

were $k_{\mathrm{YdaMde}} = k_{\mathrm{cat}}^{\mathrm{R7}}$ and $(k_{-1}^{\mathrm{R7}} + k_{\mathrm{cat}}^{\mathrm{R7}})/k_1^{\mathrm{R7}} \approx k_{-1}^{\mathrm{R7}}/k_1^{\mathrm{R7}} = K_{\mathrm{d}}^{\mathrm{YdaM}}$.

S1.3 Chemical Master Equation of the curli regulation system

In order to derive a stochastic model of the curli reaction system we assumed that the underlying dynamics are governed by a state-discrete time-continuous Markov jump process [6]. The probability distribution of the molecular species vector $\mathbf{X} = [X_1, X_2, X_3]^T$ at time t is denoted $P(\mathbf{X}, t) := \mathcal{P}(X_1, X_2, X_3, \text{time} = t)$. Using the deterministic reaction rates derived above, the Chemical Master Equation (CME) of the system is derived as follows

$$\begin{aligned}
\frac{d}{dt}P(\mathbf{X},t) &= \big(\Omega\bar{V}_1(X_1-1) + \Omega\bar{V}_4(X_3)\big)\cdot P(X_1-1,X_2,X_3,t) \\
&+ \big(\Omega\bar{V}_2(X_1+1) + \bar{V}_3(X_1+1,X_2)\big)\cdot P(X_1+1,X_2,X_3,t) \\
&+ \bar{V}_5(X_1,X_2+1)\cdot P(X_1,X_2+1,X_3,t) \\
&+ \bar{V}_6(X_2-1)\cdot P(X_1,X_2-1,X_3,t) \\
&+ \bar{V}_7(X_2,X_3+1)\cdot P(X_1,X_2,X_3+1,t) \\
&+ \bar{V}_8(X_3-1)\cdot P(X_1,X_2,X_3-1,t) \\
&- \Big[\Omega\bar{V}_1(X_1) + \Omega\bar{V}_2(X_1) + \bar{V}_3(X_1,X_2) + \Omega\bar{V}_4(X_3) + \bar{V}_5(X_1,X_2) \\
&+ \bar{V}_6(X_2) + \bar{V}_7(X_2,X_3) + \bar{V}_8(X_3)\Big]\cdot P(X_1,X_2,X_3,t), \qquad \text{(S11)}
\end{aligned}$$

where the bars over the reaction rates indicate a scaling of the deterministic parameters *YciRtotal*, *YdaMtotal*, $K_{\mathrm{i}}^{\mathrm{YegE}}$, $K_{\mathrm{m}}^{\mathrm{YciR}}$, $K_{\mathrm{d}}^{\mathrm{YciR}}$, $K_{\mathrm{d}}^{\mathrm{YdaM}}$, and $K_{\mathrm{d_{polymer}}}^{\mathrm{YdaM}}$ by multiplying them by the cell volume Ω.

S2 Validity of the Michaelis-Menten approximation in the deterministic and stochastic formulation

The usage of the Michaelis-Menten rates as an approximation to elementary reactions systems contains at least three potential problems. Firstly, it is not automatically ensured that the deterministic Michaelis-Menten model approximates the elementary mass action reactions sufficiently well. Furthermore, embedding a Michaelis-Menten reaction into a larger reaction system may invalidate the underlying quasi steady-state assumption. Finally, even if the deterministic reaction rates and their embedding within a larger network is valid, it is still not obvious how to formulate the Michaelis-Menten rates in a discrete stochastic model. In the following we will address all of the mentioned aspects.

Previously, using singular perturbation analysis it was shown that the Michaelis-Menten approximation is only valid in a region of the parameter

space where the following condition holds (see eq. (81) in [7], alternatively, eq. (8) in [8]):

$$E_{\mathrm{T}} \ll S_0 + K_{\mathrm{m}}. \qquad \text{(S12)}$$

In addition, the authors in [7] show that a catalytic system based on a Michaelis-Menten approximation can only be embedded within a larger reaction network if the substrate S does not appear in other fast reactions.

In order to ensure the validity of the Michaelis-Menten approximation for the reactions of the curli regulation system, derived in the sections S1.1 and S1.2 of this SI text, we added the condition (S12) as a constraint to the parameter identification procedure, described in the *Methods* section of the main manuscript. As a result, the parameters were sampled from a region where the Michaelis-Menten approximation is valid. Furthermore, the validity of the embedding of a Michaelis-Menten reaction rates within a larger network was ensured by assuming a hierarchy of the time scales of reaction rate parameters where the fast time scale of intermediate complex formation

$$t_{\mathrm{fast}} = \frac{1}{k_1(S_0 + K_{\mathrm{m}})} \qquad \text{(S13)}$$

of any reaction $R1$ - $R8$ is smaller than the time scale of product formation of any other reaction where S is involved (see eq. (13) in [7] or alternatively eq. (14) in [8]). Since for the described reactions the parameter k_1 is not known, we assumed it to be sufficiently large, so that the embedding of the Michaelis-Menten system is valid. Note that for the reactions where the additional assumption $k_1 \ll k_{-1}$ was used, it is required that the parameter k_{-1} is sufficiently large such that both conditions are fulfilled.

The validity of the Michaelis-Menten approximation within discrete stochastic models was discussed by Sanft *et al.* [8]. The authors review two different conditions for the validity of the Michaelis-Menten approximation in the stochastic context, as suggested by Rao and Arkin and Mastny *et al* [9, 10]. They conclude that the deterministic validity of the MM-rates, i.e. condition (S12), ensures the validity of either (or both) of the two conditions derived in the two studies. Thus, given that condition (S12) holds, one can generate a stochastic propensity function from the deterministic MM-rate (see e.g. eq. (S2)) by using corresponding discrete molecular numbers instead of molecular concentrations.

References

[1] Klipp, E., Herwig, R., Kowald, A., Wierling, C., Lehrach, H.: Systems Biology in Practice. Wiley-VCH, Weinheim, Germany (2006)

[2] Chan, C., Paul, R., Samoray, D., Amiot, N.C., Giese, B., Jenal, U., Schirmer, T.: Structural basis of activity and allosteric control of diguanylate cyclase. Proceedings of the National Academy of Sciences of the United States of America **101**(49), 17084–17089 (2004)

[3] Christen, B., Christen, M., Paul, R., Schmid, F., Folcher, M., Jenoe, P., Meuwly, M., Jenal, U.: Allosteric Control of Cyclic di-GMP Signaling. Journal of Biological Chemistry **281**(42), 32015–32024 (2006)

[4] Christen, M., Christen, B., Folcher, M., Schauerte, A., Jenal, U.: Identification and Characterization of a Cyclic di-GMP-specific Phosphodiesterase and Its Allosteric Control by GTP. Journal of Biological Chemistry **280**(35), 30829–30837 (2005). doi:10.1074/jbc.m504429200

[5] Lindenberg, S., Klauck, G., Pesavento, C., Klauck, E., Hengge, R.: The EAL domain protein YciR acts as a trigger enzyme in a c-di-GMP signalling cascade in E. coli biofilm control. The EMBO Journal **32**(14), 2001–14 (2013)

[6] Bremaud, P.: Markov Chains: Gibbs Fields, Monte Carlo Simulation, and Queues (Texts in Applied Mathematics), Corrected edn. Springer, New York (2008). http://www.worldcat.org/isbn/0387985093

[7] Segel, L.A., Slemrod, M.: The Quasi-Steady-State Assumption: A Case Study in Perturbation. SIAM Review **31**(3), 446–477 (1989)

[8] Sanft, K.R., Gillespie, D.T., Petzold, L.R.: Legitimacy of the stochastic Michaelis–Menten approximation. IET Systems Biology **5**(1), 58–69 (2011)

[9] Rao, C.V., Arkin, A.P.: Stochastic chemical kinetics and the quasi-steady-state assumption: Application to the Gillespie algorithm. The Journal of Chemical Physics **118**(11), 4999–5010 (2003)

[10] Mastny, E.A., Haseltine, E.L., Rawlings, J.B.: Two classes of quasi-steady-state model reductions for stochastic kinetics. The Journal of chemical physics **127**(9) (2007)
