# Supplementary figures and images for: Logical-continuous modelling of post-translationally regulated bistability of curli fiber expression in Escherichia coli
Source: BMC Syst Biol. 2015 Jul 23;9:39. doi: 10.1186/s12918-015-0183-x (PMC4511525; doi:10.1186/s12918-015-0183-x)

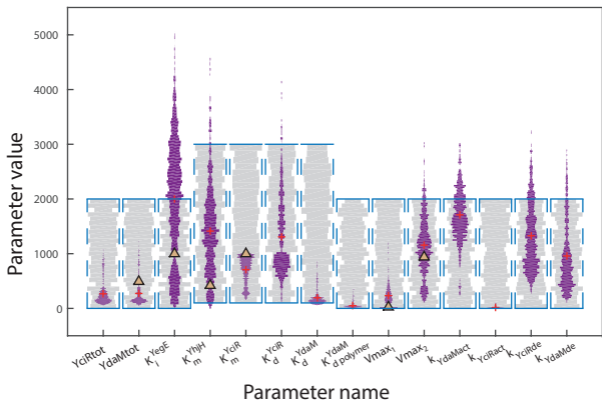

Fig. S2

Supplement: Additional file 4 — Figure S2. Violin plots showing the linearly scaled distribution of parameters in Fig. 3 of main manuscript along with the proposal distribution of the rejection sampling procedure (grey areas). The bounds of the proposal distribution, empirical means of each sampled parameter and the experimentally measured values of similar parameter values from the literature are equivalent to those in Fig. 3. [file 12918_2015_183_MOESM4_ESM.pdf]

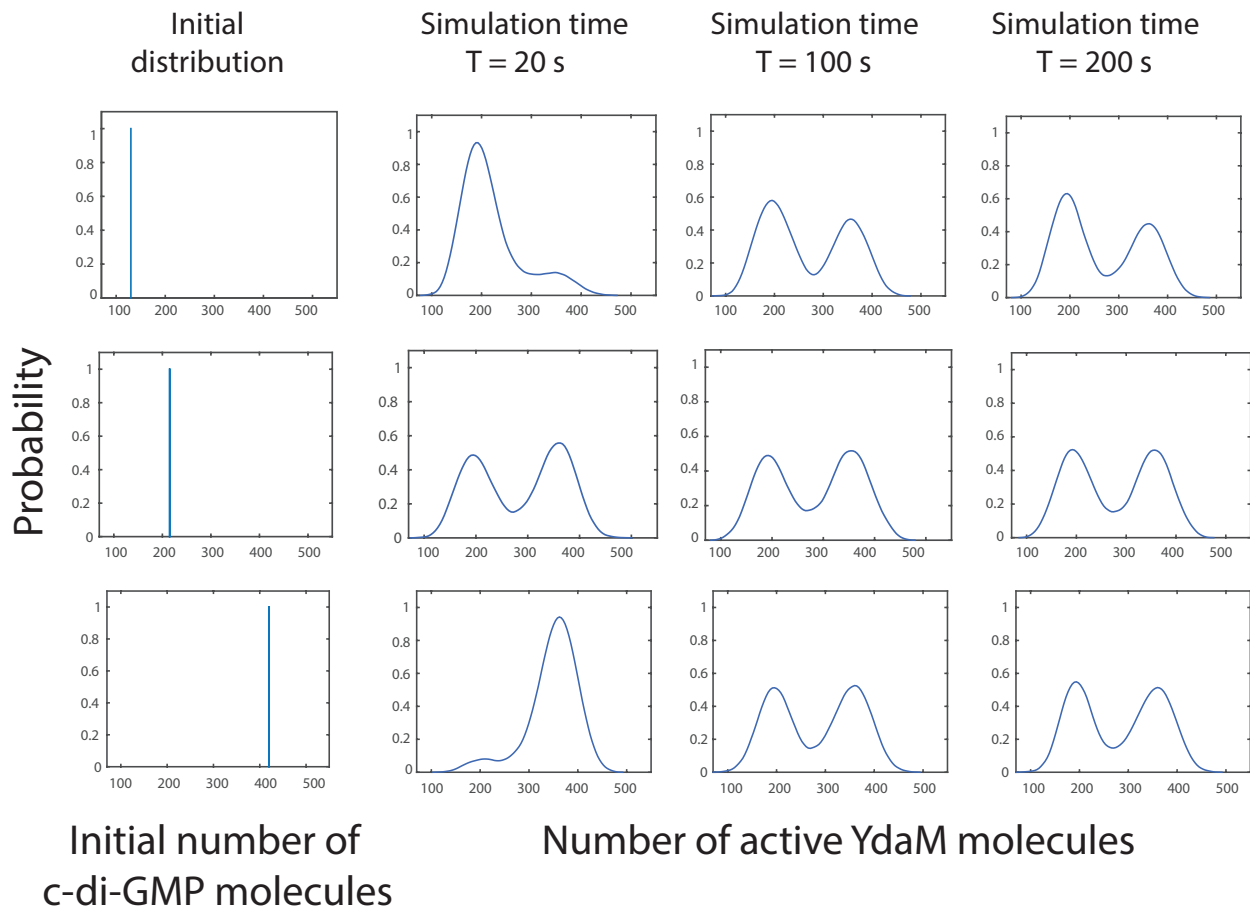

Fig. S3

Supplement: Additional file 5 — Figure S3. Solutions of the Chemical Master Equation (Additional file 2: Eq. (S11)) based on Monte Carlo sampling [42]. Three different initial levels of c-di-GMP were used: x 1=130,215 and 420 molecules (panel rows 1 to 3, respectively). The initial levels of the other two system variables were equally set to x 2=0 (YciR in YdaM/MlrA inhibition state) and x 3=768 (active YdaM). Three different simulation times T were used in order to identify the equilibration time of the system: T=20,100 and 200 seconds (panel columns 2 to 4). 103 stochastic sample trajectories were generated for computing each solution. The final empirical sampling distributions in the figures were subject to a kernel smoothing. [file 12918_2015_183_MOESM5_ESM.pdf]
